# Evaluation and manipulation of tissue and cellular distribution of cardiac progenitor cell-derived extracellular vesicles

**DOI:** 10.3389/fphar.2022.1052091

**Published:** 2022-11-24

**Authors:** Marieke T. Roefs, Wolf Heusermann, Maike A. D. Brans, Christian Snijders Blok, Zhiyong Lei, Pieter Vader, Joost P. G. Sluijter

**Affiliations:** ^1^ Laboratory of Experimental Cardiology, University Medical Center Utrecht, Utrecht, Netherlands; ^2^ IMCF Biozentrum, University of Basel, Basel, Switzerland; ^3^ CDL Research, University Medical Center Utrecht, Utrecht, Netherlands; ^4^ Circulatory Health Laboratory, Regenerative Medicine Center, University Medical Center Utrecht, University Utrecht, Utrecht, Netherlands

**Keywords:** extracellular vesicle, exosome, heart, biodistribution, targeting, endothelial cell

## Abstract

Cardiac progenitor cell-derived extracellular vesicles (CPC-EVs) have been successfully applied *via* different delivery routes for treating post-myocardial infarction injury in several preclinical models. Hence, understanding the *in vivo* fate of CPC-EVs after systemic or local, i.e. myocardial, delivery is of utmost importance for the further therapeutic application of CPC-EVs in cardiac repair. Here, we studied the tissue- and cell distribution and retention of CPC-EVs after intramyocardial and intravenous injection in mice by employing different EV labeling and imaging techniques. In contrast to progenitor cells, CPC-EVs demonstrated no immediate flush-out from the heart upon intramyocardial injection and displayed limited distribution to other organs over time, as determined by near-infrared imaging in living animals. By employing CUBIC tissue clearing and light-sheet fluorescent microscopy, we observed CPC-EV migration in the interstitial space of the myocardium shortly after EV injection. Moreover, we demonstrated co-localization with cTnI and CD31-positive cells, suggesting their interaction with various cell types present in the heart. On the contrary, after intravenous injection, most EVs accumulated in the liver. To potentiate such a potential systemic cardiac delivery route, targeting the cardiac endothelium could provide openings for directed CPC-EV therapy. We therefore evaluated whether decorating EVs with targeting peptides (TPs) RGD-4C or CRPPR connected to Lamp2b could enhance EV delivery to endothelial cells. Expression of both TPs enhanced CPC-EV uptake under *in vitro* continuous flow, but did not affect uptake under static cell culture conditions. Together, these data demonstrate that the route of administration influences CPC-EV biodistribution pattern and suggest that specific TPs could be used to target CPC-EVs to the cardiac endothelium. These insights might lead to a better application of CPC-EV therapeutics in the heart.

## 1 Introduction

Myocardial infarction (MI) is one of the main causes of mortality and the most common cause of chronic heart failure worldwide (Virani et al., 2020). Acute mortality post-MI has decreased due to improved reperfusion strategies. Still, these also induce additional damage, known as ischemia-reperfusion injury (IRI), further contributing to progressive deterioration of cardiac pump function (Algoet et al., 2022). Currently, no curative treatment is available for chronic heart failure patients besides heart transplantation (Ibanez et al., 2018). In the past years, extracellular vesicles (EVs) have been discovered to be the functional part of the progenitor cell secretome and have emerged as a promising therapeutic approach in the treatment of chronic heart failure (Timmers et al., 2007; Timmers et al., 2011; Arslan et al., 2013; Chen et al., 2013; Ibrahim et al., 2014).

EVs comprise a heterogeneous group of lipid bilayer-enclosed vesicles that have been implicated to play a role in intercellular communication in many physiological and pathological processes (Jeppesen et al., 2019). Due to their ability to transfer bioactive cargo, composed of a wide variety of nucleic acids, proteins and lipids, to target cells and tissues, they exhibit promising therapeutic features for cardiac repair (van Niel et al., 2018). Several studies demonstrated that administration of EVs derived from cardiac progenitor cells (CPCs) or closely related cardiosphere-derived cells (CDCs) was beneficial in preclinical models of acute MI or IRI, independent of the route of administration (Barile et al., 2014; Barile et al., 2018; Maring et al., 2019; Yang et al., 2019). Although high local levels of EVs can be administered to the heart with intramyocardial injection, this might not necessarily result in high EV retention as shown, for example, for stem and progenitor cells that are quickly removed from the heart upon intramyocardial administration (van den Akker et al., 2016). The retention and biodistribution of CPC-EVs after cardiac administration are largely unknown. Less invasive intravenous administration is clinically better applicable and higher volumes and multiple dosing regimens can be applied. However, unmodified EVs are rapidly cleared from the blood circulation and are mainly distributed to the liver and other organs of the reticuloendothelial system after intravenous injection in mice (Takahashi et al., 2013; Lai et al., 2014; Smyth et al., 2015; Wiklander et al., 2015; Mentkowski and Lang, 2019). Investigating the retention and biodistribution of CPC-EVs after different routes of administration is essential for the clinical application of CPC-EVs as regenerative therapy in the heart. Moreover, most EVs demonstrate limited tropism to a specific cell type (Hoshino et al., 2015; Nolte-’t Hoen et al., 2009). Increasing specificity by targeting CPC-EVs to specific cell types in the heart could provide additional features apart from their low immunogenicity, biological tolerability, and ability to cross multiple biological barriers, further developing them as natural therapeutics or drug carriers (Alvarez-Erviti et al., 2011; EL Andaloussi et al., 2013).

In this study, we employed different imaging techniques to study the short-term and long-term retention of CPC-EVs in the heart and biodistribution to other organs after intramyocardial injection and intravenous administration in mice. We, for the first time, investigated EV migration in the heart by employing CUBIC tissue clearing combined with light-sheet fluorescent microscopy and studied EV association with specific cell types resident in the heart. This improved our understanding of EV retention in the heart at tissue and cellular levels. Moreover, we sought to enhance CPC-EV targeting towards the cardiac endothelium through controlled expression of endothelial targeting peptides RGD-4C (ACDCRGDCFCG) or CRPPR to the extravesicular N-terminus of Lamp2b on the CPC-EV surface.

## 2 Materials and methods

### 2.1 Cell culture and preparation of conditioned medium

Human microvascular endothelial cells (HMEC-1) were cultured in MCDB131 medium (Invitrogen) supplemented with 10% fetal bovine serum (FBS) (Promega), 1% Penicillin/Streptomycin (P/S) (Invitrogen), 5% L-glutamine (Invitrogen), 50 nM Hydrocortisone (Sigma) and 10 ng/ml rhEGF-1 (Peprotech/Invitrogen). HEK293fT cells were cultured in Dulbecco’s Modified Eagle Medium (Gibco) supplemented with 10% FBS and 1% P/S. Cardiac progenitor cells (CPCs, donor HFH070809) were obtained from human fetal hearts as described before (Smits et al., 2009b). Human fetal heart tissue was obtained by individual permission using standard written informed consent and after approval of the ethics committee of Leiden University Medical Center (Netherlands), according to the principles outlined in the Declaration of Helsinki for the use of human subjects or tissue. CPCs were cultured in SP++ medium (66% M199 medium (Gibco), 22% EGM-2 (Lonza), 10% FBS, 1% P/S, and 1% MEM nonessential amino acids (Gibco). CPCs and HMEC-1 were cultured in flasks coated with 0.1% gelatin, and all cells were kept at 5% CO_2_ at 37°C and passaged at 80–90% confluency after digestion with 0.25% trypsin. For EV-enriched conditioned medium (CM) preparation, CPCs were cultured for 3 days until 80% confluency was reached, after which medium was replaced with basal FBS- and supplement-free M199 medium.

### 2.2 Generation of lentiviral vectors expressing targeting peptide constructs

The pcDNA5 GASTM-3-RVG-10-Lamp2b-HA plasmid was obtained from Addgene (#71295) and the GASTM-RVG cDNA sequence was replaced by the glycosylation sequence GNSTM followed by BbsI restriction sites using PCR and subsequent NEBuilder HiFi DNA assembly (New England Biolabs) according to manufacturer’s instructions. Afterward, the open-reading frame containing an N-terminal signal peptide (SP) was cloned into the pJET1.2/blunt cloning vector (Thermo Scientific) by PCR and NEBuilder HiFi DNA assembly, after which specific targeting peptide (TP) tags flanked by BbsI restriction sites were incorporated into the BbsI cut by T4 DNA ligase (New England Biolabs) according to manufacturer’s instructions. Complete SP-GNSTM-3-TP-10-Lamp2b-HA cDNA sequences were amplified by PCR to contain BamHI and NotI overhangs and cloned into the pHAGE2-EF1alpha-IRES-PuroR-WPRE lentiviral vector (Wilson et al., 2008; de Jong et al., 2020). TP tags included: TP-FLAG (DYKDDDDK), TP1 (RGD-4C; ACDCRGDCFCG), TP2 (CRPPR) and TP-9R (RRRRRRRRR). 3; 10: glycine-serine amino acid spacers. Oligo sequences are provided in Supplementary Table S1. Primer sequences are available upon request.

### 2.3 Stable CPC line generation

Lentiviral vectors containing PalmtdTomato and PalmGFP cDNA sequences were kindly provided by Prof. Xandra Breakefield (Lai et al., 2015). To generate lentiviruses, 50–60% confluent HEK293fT cells were transfected with plasmid DNA (lentiviral plasmids mixed with pCMV delta R8.2 (Addgene#12263) and pCMV-VSV-G (Addgene #8454) helper plasmids) using Lipofectamine 3000 reagent (Life Technologies) according to the manufacturer’s instructions. After 48 h, virus-containing medium was harvested, and cell debris was removed by centrifugation at 350 x *g* for 15 min and filtration using a 0.45 µm surfactant-free cellulose acetate membrane (SCFA) syringe filter (Corning). CPCs were infected with the lentivirus-containing medium and selected with Blasticidin S (ThermoFisher Scientific) to stably express PalmGFP or PalmtdTomato. To generate lines expressing specific TPs, PalmGFP stable CPC lines were infected with TP-Lamp2b lentivirus containing medium and selected with Puromycin (ThermoFisher Scientific).

### 2.4 EV isolation by ultrafiltration and size-exclusion chromatography (SEC)

CM was collected after 24 h and first centrifuged at 2000 x *g* for 15 min, followed by 0.45 µm filtration (aPES bottle top, Nalgene) to remove cellular debris. To obtain EV-enriched CM, CM was concentrated using 100 kDa molecular weight cut-off (MWCO) Amicon Ultra-15 spin filters (Merck Millipore) until 200 µl was reached. To obtain EVs, CM was concentrated by Tangential Flow Filtration (TFF) using a Minimate TFF capsule with 100 kDa MWCO and subsequently loaded onto an S400 high-prep column (GE Healthcare) using an ÄKTA start system (GE Healthcare) containing a UV 280 nm flow cell. Fractions containing EVs were pooled, filtered using a 0.45 µm SCFA syringe filter (Corning), and again concentrated using a 100 kDa MWCO Amicon Ultra-15 spin filter (Merck Millipore). EVs were stored at 4°C for a maximum of 2 days until further use. GFP fluorescence was determined with a SpectraMax iD3 Multi-mode Microplate Reader (VWR).

### 2.5 Fluorescent EV labeling and EV purification

For *in vivo* tracking studies, CPC-EVs were labeled with Alexa Fluor 790- or Alexa Fluor 647 NHS ester dyes (Thermo Fisher Scientific). EVs were incubated with 0.05 nM reactive dye in 0.1 M NaHCO_3_ in phosphate-buffered saline (PBS) and incubated for 30 min at 37°C while shaking at 450 rpm. After labeling, the free amine-reactive dye was quenched using a final concentration of 0.1 M Tris-HCl for 30 min at RT. Quenched free dye was removed using a Sepharose CL-4B column coupled to an ÄKTA start system (GE Healthcare) containing a UV 280 nm flow cell. EV-containing fractions were concentrated using a 100 kDa MWCO Amicon Ultra-4 spin filter (Merck Millipore). Fluorescent labeling efficiency was determined by diluting different EV volumes in 50 µl PBS and measuring fluorescence at 800 nm using an Odyssey M Infrared Imager (LI-COR Biosciences).

### 2.6 Proteinase K treatment

EV-containing CM was treated with or without 1% Triton-X and incubated in a final concentration of 20 μg/ml Proteinase K (Promega) for 30 min at 37°C. Proteinase K was inactivated with protease inhibitor (Roche), and CM was subsequently concentrated using 100 kDa MWCO Amicon Ultra-4 spin filters. CM samples without treatment with Proteinase K, but with the subsequent concentrating steps, were taken along in parallel and served as untreated controls.

### 2.7 EV uptake and fluorescent microscopy

For *in vitro* EV uptake experiments, HMEC-1 (1.4 × 10^4^/well) were seeded on cover glass in a 24-wells plate and incubated for 24 h (647-NHS ester-labeled) PalmtdTomato- or PalmGFP-EVs were administered to the culture media of the recipient cells and incubated for 4 h at 37°C, after which HMEC-1 were washed with PBS and fixed using 4% paraformaldehyde (PFA). Cells were stained with Alexa Fluor 555 or -488 phalloidin (Thermo Fisher Scientific), and nuclei were stained using 1:10,000 Hoechst 33342 (Invitrogen) for 30 min at RT. The cover glass was mounted on a slide using Fluoromount-G (SouthernBiotech) or Mowiol and fluorescent images were taken using a Thunder fluorescent microscope (Leica).

### 2.8 Genomic DNA extraction and PCR

Genomic DNA was extracted from stable CPC lines using the GeneJet Genomic DNA Purification kit (Thermo Scientific) following the recommended protocol. To assess successful incorporation of the targeting peptide constructs in the CPC genome, the construct-specific cDNA was amplified by PCR using Q5 Hot Start High-Fidelity 2x Master Mix (NEB) on 100 ng DNA template. After an initial incubation at 98°C for 30 s, 35 cycles of 98°C for 10 s, 65–68°C for 30 s, and 72°C for 30–45 s were used followed by a final extension at 72°C for 10 min. Annealing temperature and extension time was dependent on the used primer sequences, which can be found in Supplementary Table S2. Primers amplifying ß-actin were included as a control to validate the presence of genomic DNA. PCR products were separated on a 1% agarose gel containing ethidium bromide, and gels were imaged with a Gel Doc XR + imaging system (Bio-Rad) to confirm the presence of the constructs.

### 2.9 Nanoparticle tracking analysis

EV particle size distributions were produced using a Nanosight NS500 system (Malvern Technologies) equipped with a 405 nm laser, capturing three videos of 30 s using a camera level of 16 and a detection threshold of 5. EVs were diluted in PBS to a concentration of between 30 and 100 tracks per frame. Size and particle concentration were determined with the Nanosight NTA 3.3 software (Malvern Technologies).

### 2.10 EV protein determination

Total protein concentrations of EV samples were determined using the Pierce microBCA Protein Assay Kit (Thermo Fisher Scientific) according to the manufacturer’s protocol after lysis in 1x RIPA buffer (Abcam).

### 2.11 Western blot for EV markers

EV samples were mixed with NuPAGE sample reducing agent (Thermo Fisher Scientific) and NuPAGE sample buffer (Thermo Fisher Scientific), heated to 90°C for 10 min, and equal amounts of proteins were subjected to electrophoresis over 4–12% Bis-Tris polyacrylamide gels (Thermo Fisher Scientific). Proteins were blotted on Immobilon-FL polyvinylidene difluoride (PVDF) membranes (Merck Millipore), which were subsequently blocked with 50% v/v Odyssey Blocking Buffer (LI-COR Biosciences) in Tris-buffered saline (TBS). Antibodies were incubated in 50% v/v Odyssey Blocking Buffer in TBS containing 0.1% v/v Tween 20 (TBS-T). Primary antibodies used were rabbit anti-Calnexin (GeneTex, GTX 101676, 1:1,000), mouse anti-β-actin (Sigma, 1:5,000), mouse anti-Syntenin-1 (Origene, TA504796, 1:1,000), rabbit anti-Annexin A1 (Abcam, ab214486, 1:1,000), mouse anti-CD81 (clone B-11, Santa Cruz, 1:1,000), rabbit anti-GAPDH (clone 14C10, Cell Signaling, 1:2,000), goat anti-HA (GenScript, A00168, 1:1,000), rabbit anti-RFP (Rockland Immunochemicals, 600-401-379, 1:1,000), and mouse anti-GFP (clone GF28R, Thermo Fisher Scientific, MA515256, 1:1,1000). Secondary antibodies included Alexa680-conjugated goat anti-mouse (Thermo Fisher Scientific, 1:7,500) and IRG800-conjugated goat anti-rabbit (LI-COR Biosciences, 1:7,500). Imaging was performed on an Odyssey M Infrared Imager (LI-COR Biosciences) at 700 and 800 nm.

### 2.12 Transmission electron microscopy (TEM)

Concentrated EVs were adsorbed to carbon-coated formvar grids for 15 min at RT. After a PBS wash, the grids were fixed in a 1% glutaraldehyde in PBS fixing buffer for 30 min at RT, followed by counterstaining with uranyl-oxalate. Grids were embedded in a mixture of 1.8% methylcellulose and 0.4% uranyl acetate at 4 °C and imaged on a Jeol JEM-1011 TEM microscope (Jeol).

### 2.13 HMEC-1 uptake experiments

200,000 HMEC-1 were plated 1 day, or 100,000 HMEC-1 were plated 2 days before stimulation in a 24-wells plate or µ-Slide I 0.6 Luer (Ibidi). The day before stimulation, µ-slides were connected to a yellow/green Ibidi Perfusion Set (Ibidi) and HMEC-1 were subjected to a 300/s shear rate to mimic the shear stress in the venous system employing the Ibidi Pump System connected to the PumpControl Software (Ibidi), according to manufacturer’s instructions. Before stimulation, HMEC-1 were supplemented with fresh complete growth medium and concentrated CM was administered, normalized on total GFP fluorescence. Empty M199 medium was supplemented as a negative control. µ-slides were subjected to 300/s shear rate. For peptide inhibition experiments, 50 µM recombinant peptides (NH2-CRGDC-COHN2 or NH2-CRPPR-CONH2; DGpeptides) were added to µ-slides and subjected to a 300/s shear rate 30 min before CM addition. After 4 h, HMEC-1 were harvested employing trypsinization and EV uptake was analyzed using a CytoFlex flow cytometer (Beckman) for GFP expression.

### 2.14 *In vivo* tracking experiments

All animal experiments were performed at the University Utrecht in compliance with ‘Guide for the Care and Use of Laboratory Animals’ and were approved by the Animal Ethical Experimentation Committee, Utrecht University, Netherlands. We used surplus BALB/c or C57BL/6 mice (male or female, 10–14 weeks old) and animals received standard chow and water *ad libitum* and were housed under standard conditions with 12 h light/dark cycles until experimental procedures. Mice were anesthetized by intraperitoneal injection of medetomidine (0.05 mg/kg body weight), midazolam (5 mg/kg) and fentanyl (0.5 mg/kg), followed by intubation and mechanical ventilation (1:1 oxygen-air ratio). An automatic heating blanket-maintained body temperature at 37°C during surgery. Left lateral open thoracotomy was performed and followed by intramyocardial (IM) injection of 5 µl CPC-EVs in the left ventricle using a 30G needle. Next, surgical wounds were closed, followed by a subcutaneous injection of antagonists consisting of atipamezole hydrochloride (1.0 mg/kg), flumazenil (0.5 mg/kg), and buprenorphine (0.1 mg/kg). Mice were euthanized with an intraperitoneal injection of pentobarbital (150 mg/kg). For short-term near-infrared imaging (NIRF) imaging experiments, male C57BL/6 mice were injected with 6 × 10^9^ 790NHS ester-labeled EVs followed by NIRF imaging up to 20 min after injection. For long-term *in vivo* tracking experiments, male BALB/c were injected with 4 × 10^9^ 790NHS ester-labeled EVs followed by NIRF imaging up to 5 days after injection. 50 µL containing 4 × 10^9^ 790NHS ester-labeled EVs in PBS were injected in the tail vein for 90 min follow-up after intravenous injection. For EV-cell association experiments, 1 × 10^10^ PalmtdTomato^+^ EVs were IM administered in male Balb/c and C57BL/6 mice. After 4 h, the heart was perfused with PBS and EV association to specific cells was determined by immunohistochemistry. To determine EV distribution in the myocardium by whole-mount tissue clearing, 2 × 10^10^ PalmTdTomato^+^ 647NHS ester-labeled EVs were administered to the heart of female C57BL/6 and BALB/c mice by IM injection. 20 min after administration, the heart was perfused with 10 μg/ml Lectin-FITC (Merck) in PBS, followed by perfusion and storage in 4% PFA.

### 2.15 Near-infrared fluorescent (NIRF) imaging

The localization and bio-distribution of 790-NHS-Esther- and DiR-labeled EVs were examined using NIRF imaging in mice at various time points after intramyocardial EV administration. Images were acquired on a PearlTM Impulse Imaging system (LI-COR Biosciences) in living animals before and immediately following EV administration. At the final time point, mice were terminated, blood was collected from the retro-orbital plexus, and individual organs were imaged.

### 2.16 Tissue lysis and fluorescent measurement

At termination, individual organs were collected, snap-frozen and stored at −80°C. Organs were weight, cut, and lysed in 1x RIPA buffer (Abcam) using ceramic beads and a Precellys 24 tissue homogenizer (Bertin Instruments). Total fluorescence of 50 µl lysed sample was measured using an Odyssey M Infrared Imager (LI-COR Biosciences) at 800 nm. Fluorescent units were determined as mean grey value, corrected for background fluorescence of organs collected from the negative control (PBS-injected) mice.

### 2.17 Immunocytochemistry

Post-euthanasia, organs were collected and cut in half across the infarct. Half was used for cryosectioning and tissue lysis for total fluorescent measurements. The other half was fixed in 4% PFA and embedded in paraffin, followed by confocal microscopy. In short, heart sections (4 μm) were cut, deparaffinized and exposed to heat-induced epitope retrieval in 10 mM citrate buffer (pH 6.0) followed by 10% goat serum blocking. Sections were stained overnight at 4°C with rabbit anti-human RFP (Rockland, 600-401-379, 1:500) in 1% BSA in PBS, followed by incubation with poly-HRP-conjugated secondary antibody. Signals were amplified using the Alexa Fluor 555 Tyramide Signal Amplification kit (Thermo Fisher Scientific, B40923), according to the manufacturer’s instructions. Slides were then extensively washed in PBS and incubated for 1 h with rabbit anti-human PECAM-1 (Santa Cruz, sc-1506, 1:100) or rabbit anti-human TNNI3 (Santa Cruz, sc-15368, 1:100), followed by incubation with anti-rabbit secondary antibody conjugated to Alexa 488 (Thermo Fisher Scientific, A11034, 1:400). DAPI (1 mg/ml) was added for 15 min and slides were mounted using Mowiol. Fluorescent signal was analyzed by a Thunder fluorescent microscope (Leica) and laser intensities and gains were set using the negative control (PBS injected) heart tissue to minimize background fluorescence. Once the laser intensity for FITC and APC was assessed using the negative control, it was kept constant across all subsequent imaging. Serial scanning was performed to prevent ‘bleed-through’ from one laser wavelength to another.

### 2.18 Whole mount tissue clearing and light-sheet fluorescent microscopy

Tissue clearing was performed using the water-based clearing method CUBIC L as described previously (Tainaka et al., 2018). In our hands, this method has been shown to preserve the fluorescence as well as the tissue structure, decolor residual blood and efficiently clear the tissue for imaging. Briefly, samples were cleared with CUBIC L solution (10% N-butyldiethanolamine (v/v), 10% Triton X-100 (v/v) in ddH20) for 2 weeks at 37°C with gentle rocking, with refreshing the solution after 1, 3, and 7 days. Followed by PBS washing 3 times for 1 h and refractive index (RI) matching in CUBIC RA solution (45% antipyrine, 30% N-methylnicotinamide) for 1 week with gentle rocking at 37°C. After RI matching, tissue was imaged *via* dual side illumination with the Zen black software (ZEISS) in CUBIC RA solution with the RI of 1.51 on a Carl ZEISS lightsheet Z1 and 7 microscope, equipped with a Plan-Apochomat 5x/0,1NA, Clr Plan-Neofluar 5x/0.16NA and Plan-Apochromat 20x/1,0 NA detection objectives and with ×5/0.1NA foc. as well as 10x/0,2NA foc. illumination objectives. Fluorescence was excited with 488 nm, 561 nm and 640 nm lasers and detected *via* 505–545 nm BP (green), 575–615 nm BP (red) or 660 nm LP (far red) filters. Acquired tiles were sorted and dual-side illuminated tiles of center positions were fused with the Zen software and then loaded in ArivisVision4D (Arivis) for stitching, visualization and analysis.

### 2.19 Statistics

Statistical analyses of scratch assay results were performed using Prism 5.0 (GraphPad Software Inc.). The statistical difference between two groups was analyzed using an unpaired Student’s T-test. Differences between more than two groups were tested with a one-way ANOVA followed by Tukey’s HSD multiple comparison test as a post-test. Differences between EV uptake with and without synthetic peptides were tested with a paired Student’s T-test. Differences with two-tailed *p*-values < 0.05 were considered statistically significant. All results are expressed as mean ± standard deviation (SD).

## 3 Results

### 3.1 PalmtdTomato- and PalmGFP-labeled EVs can be isolated from CPC-derived conditioned medium

**FIGURE 1:**
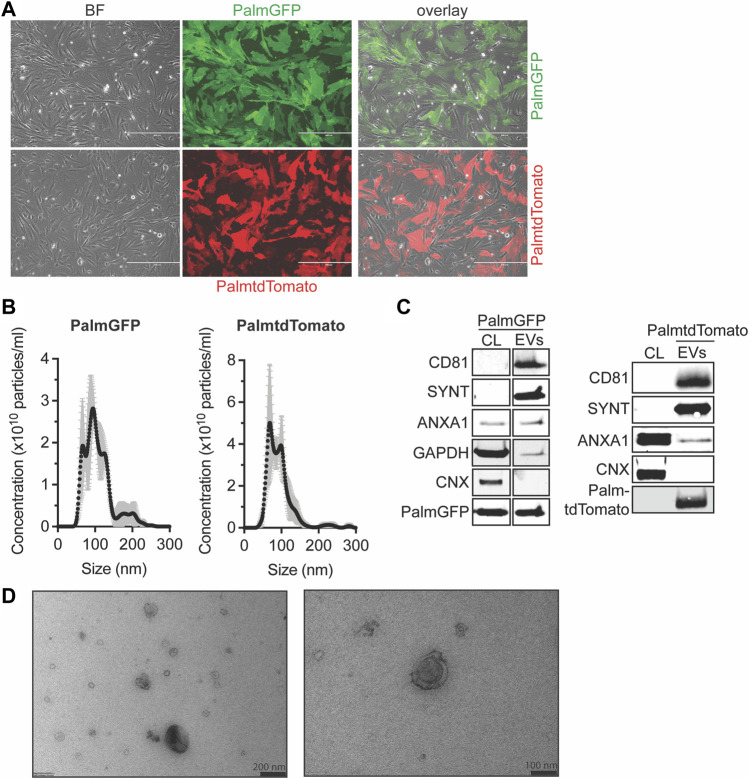
Characterization of PalmGFP- and PalmtdTomato-labeled EVs derived from CPCs. **(A)** Fluorescent microscopy pictures of CPC cells stably expressing (top) PalmGFP or (bottom) PalmtdTomato. Scale bars represent 400 µm. **(B)** Representative NTA plots showing the size distribution and particle concentration of EVs after SEC isolation of conditioned medium derived from PalmGFP^+^- and PalmtdTomato^+^ CPCs. **(C)** Western blot analysis showing the presence of CD81, Syntenin-1 (SYNT), AnnexinA1 (ANXA1), GAPDH, PalmGFP and/or PalmtdTomato, and absence of Calnexin (CNX) in (left) PalmGFP^+^- and (right) PalmtdTomato^+^ EVs. Cell lysates (CL) derived from (left) PalmGFP^+^ or (right) (TdTomato-negative) CPCs were included as control. Uncut blots are included in Supplementary Figure S10. **(D)** Representative TEM images of PalmGFP^+^ EVs at two different magnifications.

To examine EV distribution and uptake *in vitro* and *in vivo*, we labeled EVs with green fluorescent protein (GFP) or tandem dimer Tomato (tdTomato). CPCs were engineered to stably express tdTomato or GFP N-terminally fused with a palmitoylation signal (PalmGFP, PalmtdTomato) which labels cell membranes and thus EV membranes as described (Lai et al., 2015) (Figure 1A). PalmtdTomato-expressing (PalmtdTomato^+^) and PalmGFP-expressing (PalmGFP^+^) CPCs were cultured in serum-free medium for 24 h and EVs were isolated from the conditioned medium employing size-exclusion chromatography (SEC). The size distribution of the isolated EVs was determined by nanoparticle tracking analysis (NTA) and showed typical EV size distributions with median sizes of approximately 100 nm (Figure 1B). Western blot analysis showed the presence of PalmtdTomato or PalmGFP together with EV marker proteins CD81, Syntenin-1 and AnnexinA1 in PalmtdTomato^+^- and PalmGFP^+^ EVs, respectively. Calnexin was not detected in either EV population (Théry et al., 2018) (Figure 1C). TEM analyses confirmed the presence of membrane-enclosed particles of varying sizes (Figure 1D). Intravesicular membrane labeling with the constructs was confirmed as Proteinase K treatment did not affect PalmGFP expression levels while decreasing the expression of CD81 and β-actin, which are present on the surface or outside of EVs (Supplementary Figure S1). To investigate *in vitro* uptake, human microvascular endothelial cells (HMEC-1) were incubated with labeled EVs for 4 h. Fluorescent imaging demonstrated the presence of EVs in HMEC-1, which showed that both PalmGFP^+^ and PalmtdTomato^+^ EVs are being internalized (Supplementary Figure S2).

### 3.2 CPC-EVs distribute in the heart after intramyocardial injection

**FIGURE 2:**
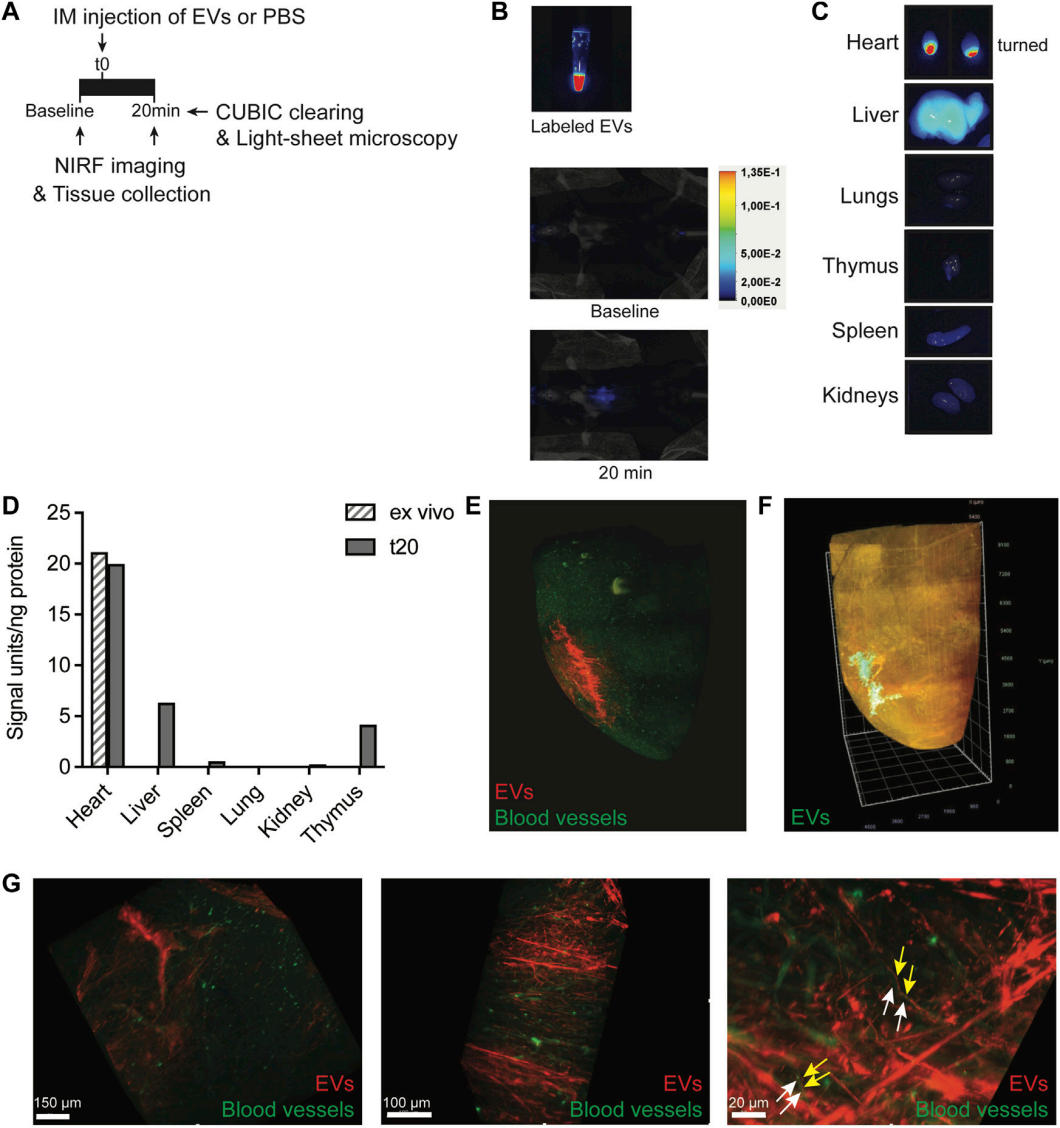
Short-term EV biodistribution after intramyocardial injection. **(A)** Schematic overview of EV administration and near-infrared (NIRF) imaging directly or at 20 min after intramyocardial injection (IM) of 5 μL EVs in the left ventricle. **(B)** NIRF images taken from the Eppendorf containing labeled EVs and living animal at baseline and 20 min after intramyocardial EV administration. **(C)** NIRF images of individual organs collected after termination. Uncut images are included in Supplementary Figure S11. **(D)** Quantification of fluorescence (800 nm) per ng protein in organ lysates as compared to organ background. An *ex vivo* heart injected with 5 μL EVs was included as control. **(E,F)** Snap-shots of 3D fluorescent images of the heart injected with **(E)** 2.5 × 10^10^ or **(F)** 1.75 × 10^10^ AlexaFluor647 NHS ester-labeled EVs, generated after CUBIC tissue clearing and subsequent light-sheet fluorescent microscopy. Images represent two independent experiments. **(E)** Heart was perfused with Lectin-FITC to stain blood vessels before tissue collection. Snapshots at higher magnification are displayed in **(G)**. Arrows indicate no co-localization of (yellow) EVs and (white) blood vessels.

Progenitor cells demonstrate rapid flush-out from the heart and display low engraftment levels after intramyocardial administration, limiting their clinical efficiency in treating post-MI injury (van den Akker et al., 2016). The retention and biodistribution of EVs directly after cardiac administration are largely unknown. Investigating CPC-EV retention and biodistribution upon intramyocardial injection in mice could provide hints for a better clinical application of CPC-EVs as a cardioprotective therapeutic. To monitor EV distribution in living animals by near-infrared (NIRF) imaging, isolated CPC-EVs were labeled with AlexaFluor790-NHS ester and subsequently purified employing SEC (Supplementary Figure S3A). NHS ester-labeled EVs demonstrated the expression of commonly used EV marker proteins (Supplementary Figure S3B) and showed a mean size and mode size of 94.1 nm and 83.6 nm, respectively (Supplementary Figure S3C). Fluorescent labeling with AlexaFluor790-NHS ester was confirmed (Supplementary Figure S3D, Figure 2B). To better understand short-term EV retention and distribution in the heart after intramyocardial injection, we administered 5 µl containing 6 × 10^9^ 790NHS ester-labeled EVs in the left ventricle wall during open-chest surgery. NIRF imaging of living animals confirmed the presence of EVs in the mouse heart 20 min after a single dose administration (Figures 2A,B). After termination, we determined the fluorescent signal in individual organs (Figure 2C) and tissue lysates (Figure 2D). A large proportion of the EV signal was still present in the heart, reaching similar levels as compared to an *ex vivo* injected heart, and limited distribution to other organs could be observed 20 min after administration.

To assess EV distribution at the tissue level, AlexaFluor647 NHS ester-labeled PalmTdTomato^+^ EV distribution was evaluated in the heart through CUBIC tissue clearing, followed by light-sheet fluorescent microscopy and 3D-reconstruction (Tainaka et al., 2018). 3D-image processing demonstrated the presence of EVs in the left ventricle wall of the mouse heart and migration from the fluid injection site into the myocardium, 20 min after injection (Figures 2E,F; Supplementary Movie S1). Before tissue collection, the heart was perfused with Lectin-FITC to stain blood vessels. Higher magnifications demonstrate that EVs did not colocalize with vessels and aligned next to the vessels (Figure 2G; Supplementary Movie S2), which hints toward the presence of EVs in the interstitial space some minutes after injection. To confirm true EV visualization and rule out the presence of free AlexaFluor647 dye, co-localization of AlexaFluor647 and tdTomato was confirmed in the *ex vivo* heart and after uptake in HMEC-1 (Supplementary Figure S4). These results imply limited flush-out of EVs from the heart and their diffusion through the interstitial space shortly after intramyocardial administration.

### 3.3 CPC-EVs are retained in the mouse heart after intramyocardial injection

Intramyocardial administration of CPC-EVs has been reported to reduce infarct size and promote cardiac function in several preclinical models several days up to weeks after administration (Barile et al., 2014; Barile et al., 2018; Maring et al., 2019; Yang et al., 2019). A large body of evidence suggests that CPC-EVs modulate different short-term and long-term cardiac repair processes, such as modulating cardiomyocyte apoptosis, promoting angiogenesis, and modulating fibrosis and the immune response upon intramyocardial injection (Sluijter et al., 2018). Investigating the retention and distribution of CPC-EVs up to days after administration might hint at whether EVs are still resident in the heart at those moments and modulate the more chronic repair processes or whether effects are mainly evoked in the short-term.

**FIGURE 3:**
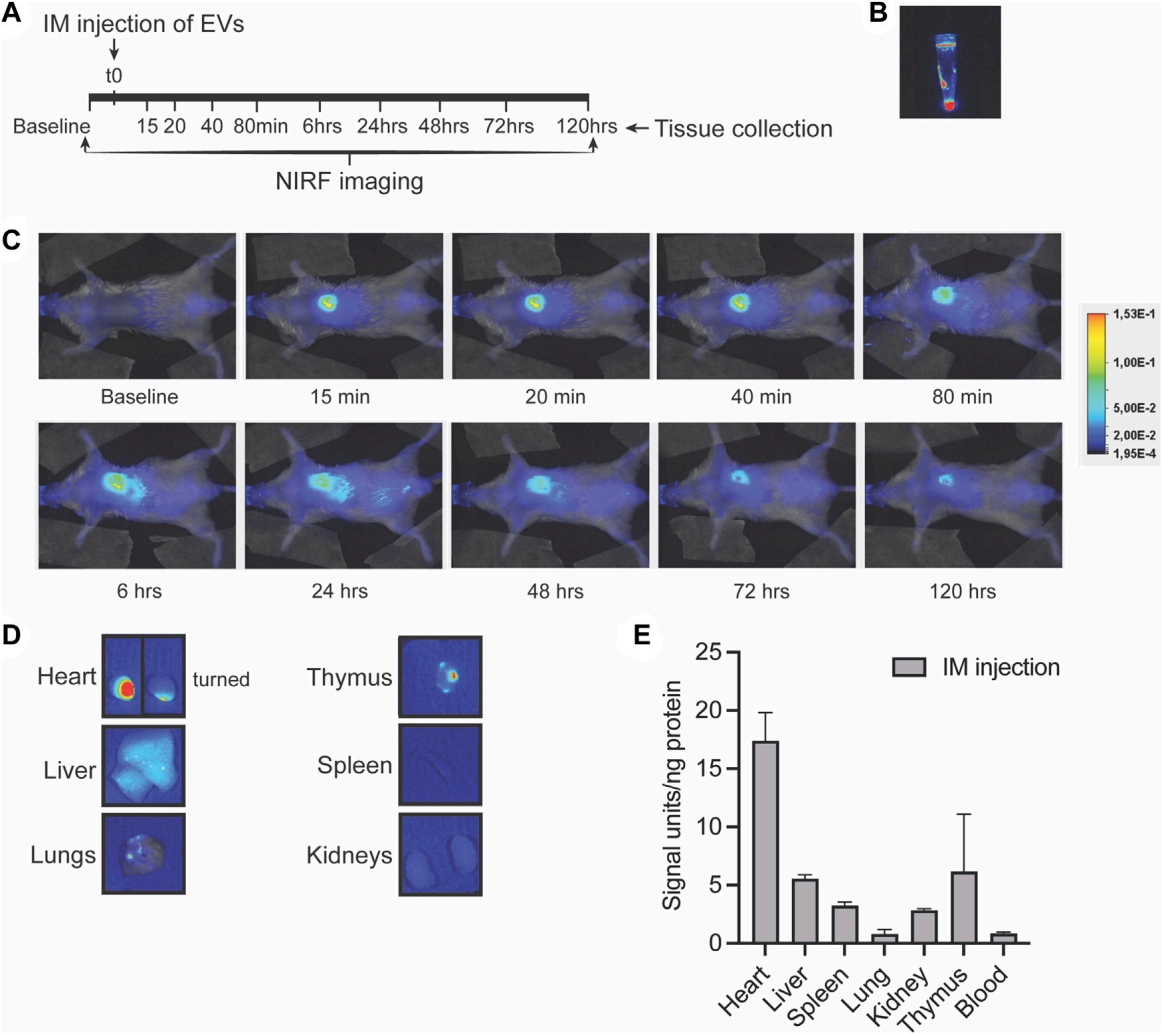
Long-term EV distribution in the mouse after intramyocardial injection. **(A)** Schematic overview of intramyocardial (IM) EV administration in the left ventricle wall and near-infrared (NIRF) imaging up to 5 days after intramyocardial injection. **(B,C)** Representative NIRF images taken from **(B)** the eppendorf containing labeled EVs, **(C)** the living animal at various time points after EV administration and **(D)** of individual organs collected after 5 days follow-up. Uncut images are included in Supplementary Figure S11. **(E)** Quantification of fluorescence (800nm) per ng protein in organ lysates as compared to organ background. Data of two mice are shown and are displayed as mean ± SD.

To assess long-term EV distribution in mice, we administered a single dose of 4 × 10^9^ 790NHS ester-labeled EVs in the left ventricle wall and followed NIRF signal distribution in living mice at different time points after injection (Figures 3A,B). Over time, an increased fluorescent signal could be detected in the liver, while a decreased but detectable fluorescent signal was retained in the heart even 5 days after injection (Figures 3B,C). On day 5, individual organs were imaged (Figure 3D) and organ lysates were analyzed for fluorescence (Figure 3E), which showed fluorescent signals in the heart and the presence of signals in the liver, spleen, lung, kidney and thymus. These findings indicate that a large proportion of EVs are retained in the heart after intramyocardial injection. This may suggest that EVs could exert therapeutic effects both immediately as well as up to days after administration.

### 3.4 CPC-EVs associate with specific cell types in the heart

**FIGURE 4:**
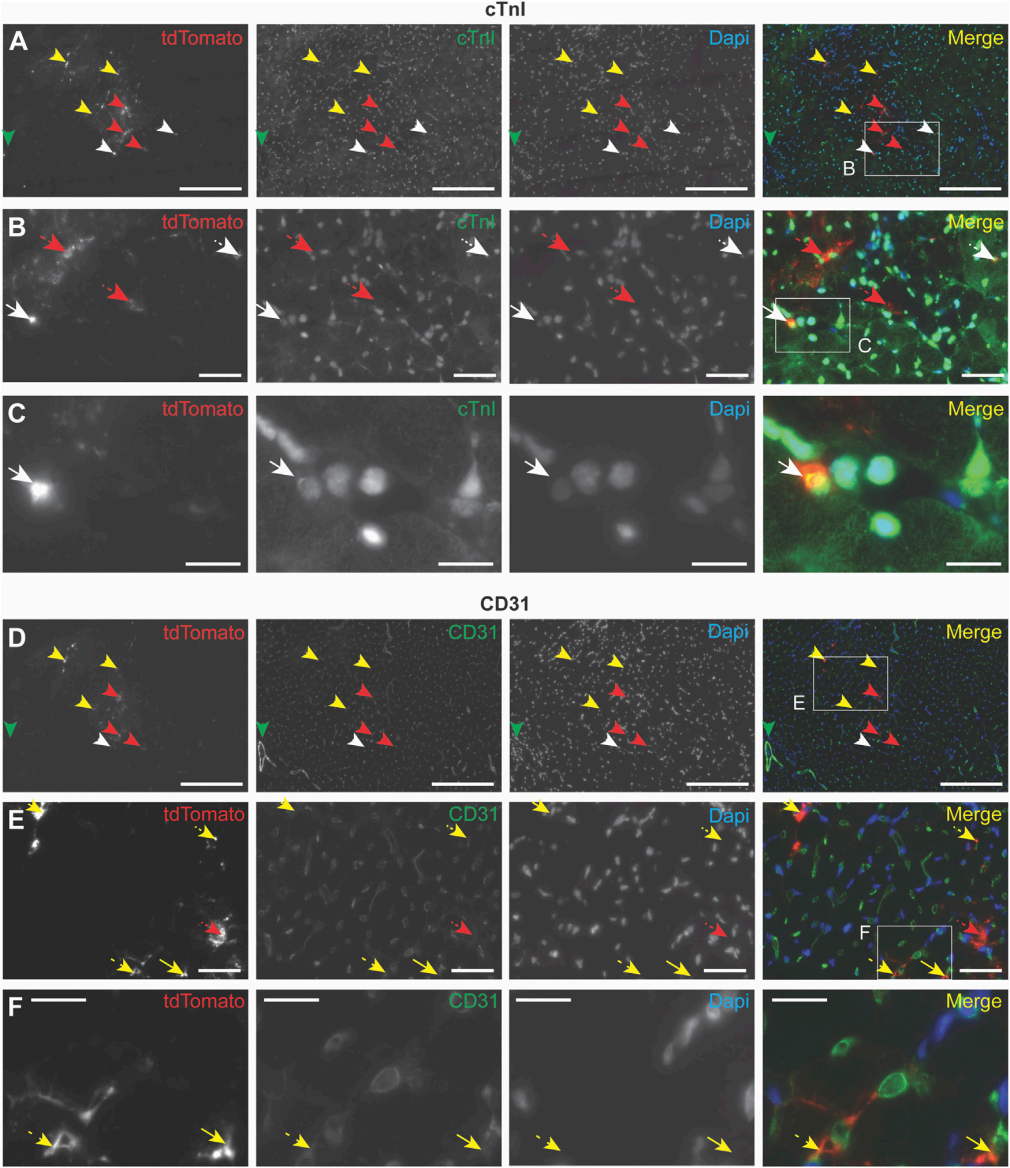
Immunocytochemistry analysis of EV uptake in the heart. PalmtdTomato-EVs were administered in the left ventricle wall of a healthy mouse heart through intramyocardial injection and heart tissue was collected after 4 h **(A–F)** Immunofluorescence staining of two subsequent heart sections, cut across the sagittal plane, using antibodies against tdTomato (shown in red), and co-staining with antibodies against **(A–C)** cardiomyocyte marker cardiac Troponin I (cTnI, shown in green) and **(D–F)** blood vessel-specific CD31 (shown in green). **(B)** Enlargement of the square in panel A. **(C)** Enlargement of the square in panel B. **(E)** Enlargement of the square in panel D. **(F)** Enlargement of the square in panel E. Nuclei are visualized with DAPI (shown in blue). tdTomato co-localization with other stainings are indicated with arrows: cTnI (white), CD31 (yellow), no co-localization (red). Scale bars = 50 μm **(A,D)**, 250 μm **(B,E)**, 100 μm **(C,F)**.

To further investigate the cell types with which EVs interact after intramyocardial injection in mice, PalmtdTomato^+^ EVs were administered in the left ventricle wall of healthy living mice. After 4 h, the heart was perfused with PBS and EV association to specific cells was determined by immunohistochemistry. Due to the weak fluorescent signal of EV-associated TdTomato, antibodies against TdTomato were used to amplify EV signal, which demonstrated the presence of EVs in the area of injection (Figures 4A–F; Supplementary Figures S5A–F). Co-staining with antibodies against cardiomyocyte-specific cardiac Troponin I (cTnI) (Figures 4A–C; Supplementary Figure S5A–C), or endothelial cell-specific CD31 (Figures 4D–F; Supplementary Figures S5D–F) showed EV co-localization with these cell types, as well as their presence in the interstitial space between cardiac cells. These results imply EV interaction with different cardiac cell types, as well as their presence in the interstitial space 4 h after intramyocardial injection.

### 3.5 CPC-EVs distribute mainly to the liver after intravenous administration

**FIGURE 5:**
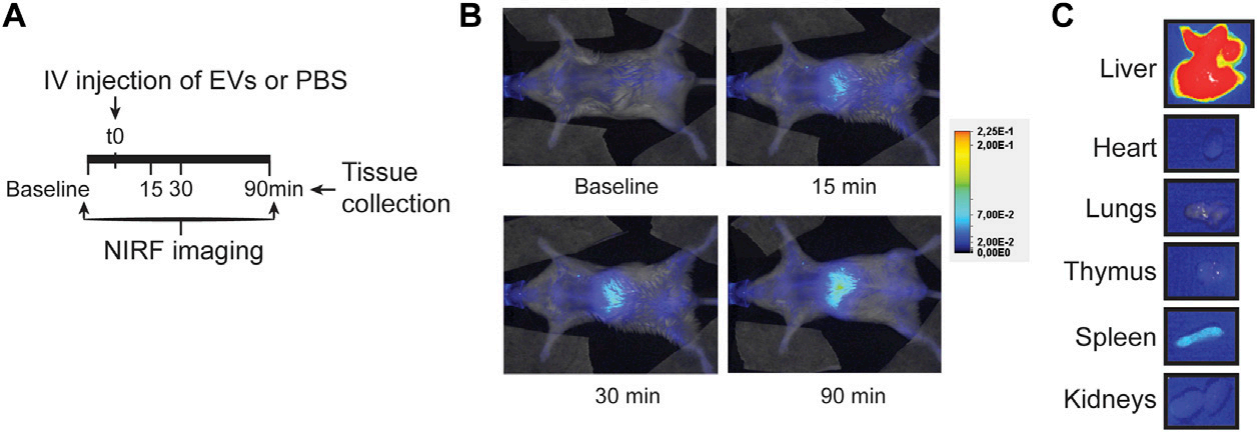
EV distribution in the mouse after intravenous injection. **(A)** Schematic overview of EV administration and NIRF imaging up to 90 min after tail vein injection. **(B,C)** Representative NIRF images taken **(B)** from the living animal at various time points after EV administration and **(C)** of individual organs collected after 90 min follow-up. Uncut images are included in Supplementary Figure S11.

An alternative, more clinically applicable method of therapeutic EV administration is through intravenous injection. This does not require open-chest surgery, and multiple dosing regimens with larger volumes can be applied. When we administered EVs in the tail vein of a healthy mouse, EVs showed a typical nanoparticle-like biodistribution pattern 90 min after injection, with EVs mainly localized in the liver while rarely in the heart (Figures 5A–C; Supplementary Figure S6). The observed limited amount of CPC-EVs in the heart after therapeutic intravenous administration corresponds with previous reports on EV distribution (Takahashi et al., 2013; Smyth et al., 2015; Wiklander et al., 2015). This suggests a need for targeted delivery of CPC-EVs to the heart to apply CPC-EV therapeutics through intravenous administration.

### 3.6 CPC-EV targeting to endothelial cells employing targeting peptides

**FIGURE 6:**
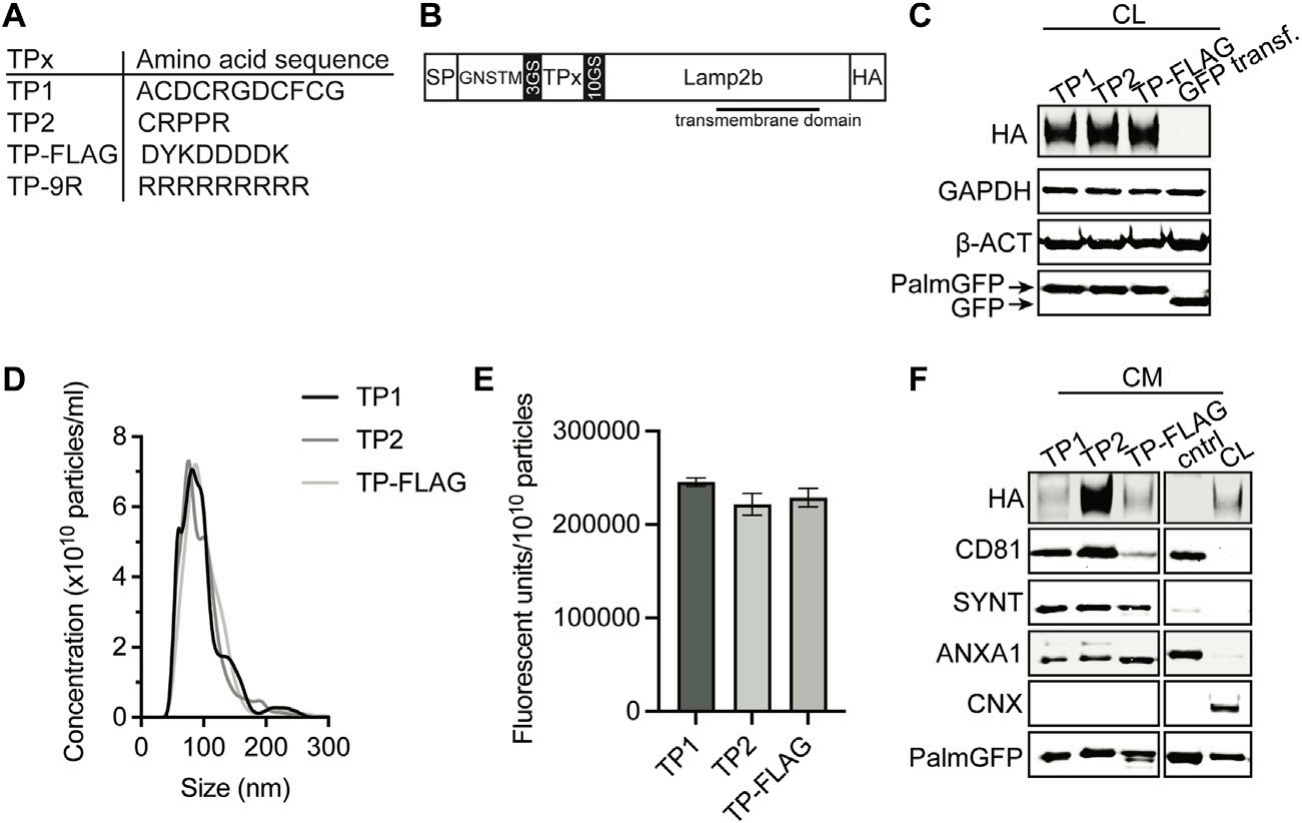
Generation of EVs expressing targeting peptides for endothelial cells. **(A)** Amino acid sequence of TP1, TP2, TP-FLAG, which was included as negative control, and TP-9R. **(B)** Schematic of constructs expressed on EVs consisting of a specific targeting peptide (TPx) connected to transmembrane protein Lamp2b, flanked by a N-terminal signal peptide (SP) and GNSTM glycosylation sequence, and C-terminal HA-tag. **(C)** Representative western blot analysis showing expression of HA-tag and PalmGFP in cell lysate (CL) harvested from CPC lines stably expressing PalmGFP^+^ and specific TPs. CPCs transduced with GFP were included as negative control. GAPDH and beta-actin (ß-ACT) were included as house-keeping proteins. **(D)** Representative NTA plots showing the size distribution and particle concentration of concentrated conditioned medium (CM) derived from PalmGFP^+^ TP-expressing CPCs. **(E)** GFP fluorescence per 10^10^ particles determined in CM derived from PalmGFP^+^ TP-expressing CPCs (n = 2). Data are displayed as mean ± SD. **(F)** Representative western blot analysis showing expression of HA-tag, PalmGFP, CD81, Syntenin-1 (SYNT) and AnnexinA1 (ANXA1) in CM. CL of PalmGFP^+^ CPCs stably expressing TP-FLAG were included as control. CM derived from CPCs transduced with GFP was included as negative control (cntrl). Calnexin (CNX) was only present in CL. Uncut blots are included in Supplementary Figure S10.

Angiogenesis is an essential process for cardiac repair, and CPC-EVs have been demonstrated to promote vessel formation *in vitro* and *in vivo* (Smits et al., 2009a; Vrijsen et al., 2010; Den Haan et al., 2012; Vrijsen et al., 2016; Mol et al., 2017; Mol et al., 2019)*.* Moreover, endothelial cells are the first cell type reached after systemic injection. Therefore, targeting EVs to the cardiac endothelium to deliver therapeutic CPC-EV cargo seems a potentially successful strategy for treating ischemia-induced chronic heart failure. One of the strategies to target specific tissues and organs is employing a display of so-called targeting peptides (TPs), which are short peptide sequences able to bind receptors on the cell membrane. Based on the literature, we selected two different endothelium-TPs for expression on the EV surface (Figure 6A). TP1 is the RGD-4C peptide (ACDCRGDCFCG) (Ellerby et al., 1999; Hölig et al., 2004), containing RGD, a ligand for αvβ3 integrins, highly expressed on activated endothelial cells (Brooks et al., 1994; Ruoslahti, 1996; Avraamides et al., 2008; Kapp et al., 2017). TP2 is peptide sequence CRPPR, previously identified by phage-display technology to target cardiac endothelium (Zhang et al., 2005). DNA sequences of these peptides were fused to the transmembrane protein lysosomal associated membrane protein 2b (Lamp2b) by insertion into a Lamp2b expression construct (Hung and Leonard, 2015), flanked by GS-linker regions, and a HA tag at the C-terminus for detection (Figure 6B). The strategy of TP fusion to Lamp2b for display on EVs has previously been employed to target the heart (Wang et al., 2018; Mentkowski and Lang, 2019), brain (Alvarez-Erviti et al., 2011; Yang et al., 2017) and tumors (Bellavia et al., 2017), but was hampered by undesired proteolytic peptide degradation in other studies (Hung and Leonard, 2015). To suppress TP cleavage from Lamp2b, we incorporated the GNSTM glycosylation motif at the N-terminus of the expression construct (Hung and Leonard, 2015). A FLAG tag and a cell-penetrating 9R peptide were included as negative and positive controls, respectively. PalmGFP^+^ CPCs were stably transduced with a lentivirus to express TP-Lamp2b. Successful incorporation of the constructs in the genome was confirmed by PCR (Supplementary Figure S7). Western blotting for the HA-tag confirmed the expression of the constructs in the CPC lysate (Figure 6C). For *in vitro* uptake experiments, EV-containing conditioned medium (CM) was collected and concentrated using 100 kDa molecular weight cut-off membranes. Particle presence in the concentrated CM was confirmed by NTA (Figure 6D), and the amount of GFP fluorescence per particle was similar across all conditions (Figure 6E). The presence of the HA-tagged construct, PalmGFP, and common EV marker proteins CD81, Syntenin-1, AnnexinA1, and absence of Calnexin was confirmed by western blotting (Figure 6F). These results demonstrated the expression of TP-Lamp2b proteins in CPC-EVs with similar characteristics among the different types of TP-EVs.

**FIGURE 7:**
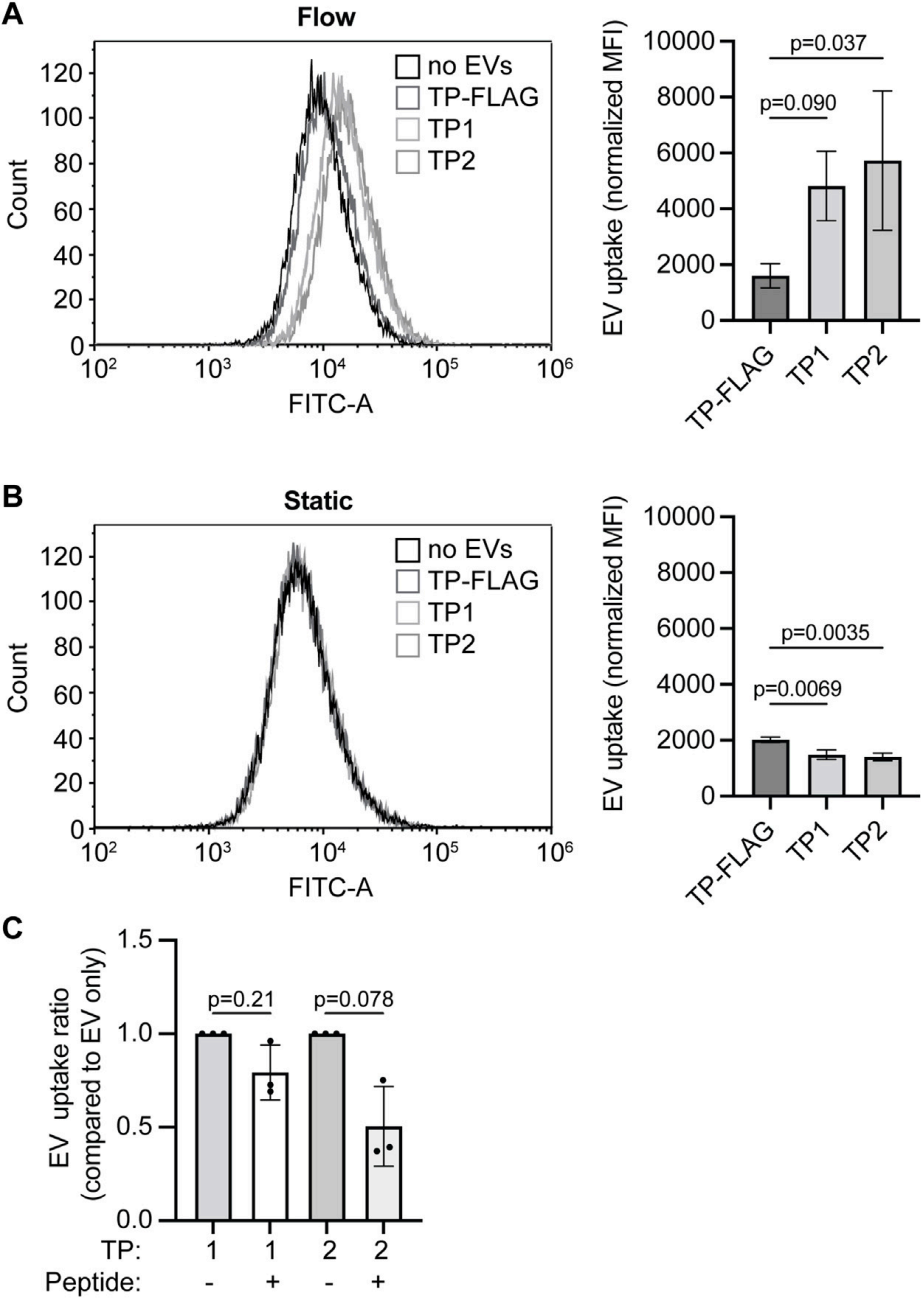
Targeting peptide expression on EVs increases EV uptake in HMEC-1 under flow conditions. **(A, B)** EV-TP1, -TP2, and -TP-FLAG uptake in HMEC-1 under **(A)** flow or **(B)** static conditions, determined by flow cytometry. Mean fluorescence is corrected for negative control (M199 medium administration). **(C)** EV-TP1 and EV-TP2 uptake in HMEC-1 under flow after HMEC-1 pre-incubation of CRGDC or CRPPR peptides. For each replicate, mean fluorescence is corrected for the average of negative control, and conditions with peptides are compared to conditions without peptide pre-incubation. Data are displayed as mean ± SD and represent three replicate experiments.

We next evaluated whether TP1 and TP2 expression enhanced EV uptake in endothelial cells *in vitro*. HMEC-1 were cultured in static 2D conditions or channel µ-slides under unidirectional continuous medium flow (at a shear rate of 300 s^−1^ comparable to human carotid arteries). EV addition was normalized on total GFP fluorescence, and EVs were incubated for 4 h under static incubation conditions or continuous medium flow. The uptake of PalmGFP^+^ particles in HMEC-1 could be confirmed by fluorescent microscopy (Supplementary Figure S8). Under continuous medium flow, uptake of EVs expressing the specific TPs exceeded uptake of TP-FLAG expressing EVs (Figure 7A), as determined by flow cytometry. For all EV types, uptake under medium flow was higher than under static conditions, as a higher fluorescent signal could be detected in HMEC-1 exposed to flow. Notably, no significant difference in HMEC-1 uptake between the specific TP-EVs and the negative control (TP-FLAG-EVs) in static conditions (Figure 7B). On the contrary, TP-9R-EVs, which were included as a positive control, demonstrated enhanced uptake (Supplementary Figures 9A–C). Uptake of TP-EVs was inhibited by preincubating HMEC-1 with synthetic CRGDC or CRPPR peptides under continuous flow, demonstrating specificity of the interaction of targeted EVs with the cells (Figure 7C). These results indicate an enhanced uptake of EVs by displaying endothelium-targeted peptides *in vitro*.

## 4 Discussion

Cardiovascular disease is the leading cause of death worldwide, and its prevalence is expected to increase in the coming decades (Virani et al., 2020). Specific and targeted cardiac repair strategies are desirable. Multiple studies have demonstrated the therapeutic potential of intramyocardial CPC-EV delivery for decreasing infarct size and improving cardiac function (Barile et al., 2014; Barile et al., 2018; Maring et al., 2019; Yang et al., 2019). However, it is known that the biodistribution of EVs influences their therapeutic efficacy and off-site effects (Mentkowski and Lang, 2019), and the actual retention and biodistribution of CPC-EVs after administration is largely unknown. In this study, we, for the first time, demonstrated that a large proportion of CPC-EVs is retained in the healthy mouse heart after intramyocardial injection. CPC-EVs were still present 20 min after injection, demonstrating no immediate flush-out of EVs from the heart. This is in line with previous observations of CDC-EV retention in the heart, 2 h after intramyocardial injection (Mentkowski and Lang, 2019). On the contrary, progenitor cells display less than 10% engraftment levels after intramyocardial injection and demonstrate rapid flush-out through clearance by venous drainage (van den Akker et al., 2016). It might be hypothesized that the smaller EVs are quickly taken up by cardiac cells or can migrate through the extracellular matrix in between cardiac cells and therefore have no direct flush-out upon cardiac administration. Indeed, by employing CUBIC tissue clearing combined with light-sheet fluorescent microscopy, we demonstrated that EVs diffuse through the interstitial space from the fluid injection site within minutes after injection.

For translational relevance, we also investigated intravenous delivery as the route of EV administration. In agreement with previous reports, CPC-EVs were distributed primarily to the liver and minimally to the heart after intravenous injection in healthy mice (Takahashi et al., 2013; Lai et al., 2014; Smyth et al., 2015; Wiklander et al., 2015; Mentkowski and Lang, 2019). Uptake of EVs in the heart is restricted by limited passing through the cardiac capillaries due to tight junctions between their endothelial cells, while after IRI, when the microvasculature gets damaged, access to the interstitial myocardium is limited due to the no-reflow phenomenon (Kloner, 2011). Therapeutic effects have been observed when EVs were administered through intravenous injections (Yang et al., 2019), which hints towards a more systemic, immune-modulating effect when EVs are applied in the circulation, while different, local cardioprotective mechanisms may be initiated after intramyocardial injection (Sluijter et al., 2018). This demonstrates the importance of discriminating between localization and function. Sites of EV accumulation might not equal areas of function and only show a final stage. Our findings demonstrate that CPC-EVs co-localize with cardiomyocytes and endothelial cells after administration in the left ventricle wall, suggesting their interaction with these cell types. This is in accordance with previous observations of CPC-EV uptake by cardiac cells in the ischemic left ventricle (Maring et al., 2019), and internalization in cardiomyocytes (Chen et al., 2013; Barile et al., 2018; Ciullo et al., 2019), endothelial cells (Youn et al., 2019), macrophages and cardiac fibroblasts (Ciullo et al., 2022) *in vitro*. EVs can activate recipient cells not only after their uptake and subsequent release of bioactive cargo, but also through direct EV-cell interactions for which no internalization is required (Roefs et al., 2020; van Niel et al., 2022). Future studies should focus on which mechanisms are involved in the active uptake and cellular activation of CPC-EVs, and if accumulation correlates with function.

Live imaging of single EVs in mice is hindered by limited tissue penetration of fluorescent and bioluminescent signals and impractical quantitative analysis, and is currently restricted to tissues immediately adjacent to mammary imaging windows or larger EVs (Zomer et al., 2015; Van Der Vos et al., 2016; Gupta et al., 2020; Verweij et al., 2021). Moreover, for lipophilic dyes, non-specific binding to contaminants in EV preparations leading to the transfer of lipophilic dyes to recipient cells was reported (Lai et al., 2015; Takov et al., 2017). We, therefore, employed fluorescent NHS-ester dyes that bind proteins present at the surface of CPC-EVs, to obtain sufficient EV labeling for CPC-EV visualization in living mice. A drawback of this labeling technique is the potential labeling of protein contaminants and the presence of reactive free dye in EV preparations. However, we observed a typical nanoparticle distribution pattern of EVs upon intravenous administration with no immediate free-dye excretion by the kidneys. Also, the co-localization of the NHS-ester dye and EV-contained PalmtdTomato demonstrates true labeling of our EV preparations and ruling out the contribution of free dye. Another danger is that the half-life of the dye greatly exceeds that of EVs and EV degradation after cellular uptake can be masked by recycling and trafficking of the fluorescent dye itself or protein fragments (Verweij et al., 2021). Since EVs are rapidly cleared from the circulation and tissue (Lai et al., 2014; Imai et al., 2015; Gupta et al., 2020), the sensitivity of our approach might be questioned at later time points and our approach might be best suited for distribution studies up to a day follow-up. Apart from the route of administration, donor cell source and modifications, EV dose and the pathological conditions of the *in vivo* model could affect EV retention and biodistribution profile (Wiklander et al., 2015). IRI affects endothelial barrier function, which could alter the efficiency of EV propagation and uptake (Verweij et al., 2021). The CPC-EV retention upon intramyocardial injection in a more clinically relevant IRI model and the influence of EV purity on CPC-EV uptake and distribution is a subject for further studies.

Although intramyocardial injection provides a higher therapeutic dose at the target site, intravenous injections might be more suitable for therapeutic applications as this technique is less invasive, there is no need for open-chest surgery, and repetitive treatments in larger volumes can be applied. However, previous reports demonstrated that <1% of EVs isolated from different cell sources accumulate in the non-injured heart upon systemic injection (Takahashi et al., 2013; Lai et al., 2014; Smyth et al., 2015; Wiklander et al., 2015; Mentkowski and Lang, 2019). Moreover, with a few exceptions, most naturally secreted EVs demonstrate limited tropism to a specific cell type (Hoshino et al., 2015; Nolte-’t Hoen et al., 2009). This reflects the need for strategies to improve CPC-EV targeting of cardiac cells. To achieve targeting of EVs to the cardiac endothelium for use in ischemic heart disease, we modified prior genetic approaches to create recombinant fusion proteins of the RGD-4C peptide (ACDCRGDCFCG) or CRPPR peptide connected to the extra-EV N-terminus of Lamp2b. Overexpression of Lamp2b fusion proteins and other protein fusion complexes has previously been demonstrated not to alter EV morphology (Tian et al., 2014; Kooijmans et al., 2016; Li et al., 2020)*.* RGD-4C is a double cyclic peptide that binds to integrin αvβ3, which is preferentially expressed on the vascular surface in the ischemic area after IRI (Brooks et al., 1994; Cleaver and Melton, 2003; Mitra et al., 2005). RGD peptide expression on the EV surface has been demonstrated to induce EV targeting to the vasculature of tumor and brain (Tian et al., 2014; Tian et al., 2018; Cao et al., 2019; Zhang et al., 2019; Xin et al., 2021; Yang et al., 2021). CRPPR has been shown to improve (cardiac) endothelium targeting of synthetic and natural systems by binding to neuropilin-1 (Zhang et al., 2005; Zhang et al., 2008; Teesalu et al., 2009; Greig et al., 2010; Kean et al., 2012; Zhang et al., 2012; Paoli et al., 2014; Zhang et al., 2015), an isoform-specific receptor for vascular endothelial growth factor, upregulated in endothelial cells after ischemic injury in the peri-infarct region (Soker et al., 1998; Zhang et al., 2001; Beck et al., 2002; Zentilin et al., 2010). It might be hypothesized that under ischemic conditions in the heart, the local upregulation of Neuropilin-1, together with a more leaky vasculature after MI induction, will lead to enhanced uptake of EVs at the target site.

In (patho)physiological conditions, the connection between EVs and endothelial cells in blood vessels is subjected to the effects of constant blood flow (i.e., shear stress). Fluid-induced shear stress induces conformational changes of endothelial cells and leads to the activation of integrin αvβ3 on the cell membrane (Tzima et al., 2001; Li et al., 2005; Hennig et al., 2011), and upregulation of neuropilin-1 mRNA expression (Thi et al., 2007). This might influence how TP-EVs interact with endothelial cells compared to static conditions frequently used in *vitro* assays that aim to study EV uptake (Van Der Meer et al., 2009; Valencia et al., 2012; Yang Chen et al., 2020). To better represent the *in vivo* EV-endothelial cell contact, we studied EV uptake under medium flow at a shear rate of 300 s^−1^, comparable to the mean shear rate reported for the human carotid artery (Reneman et al., 2006; Stroev et al., 2007). We demonstrated that under continuous medium flow, EV uptake in endothelial cells was enhanced by displaying the specific TPs. In line with our results, Wang *et al.* demonstrated increased targeting of RGD-coated EVs to blood vessels both *in vitro* and in zebrafish (Wang et al., 2017). Others demonstrated the increased interactions between HUVECs and cyclic-RGD-expressing PEGylated nanoparticles under continuous flow. However, no comparison between static and flow conditions was performed (Martínez-Jothar et al., 2020). We here directly compared *in vitro* static and flow conditions on TP-mediated EV uptake and demonstrated the importance of investigating EV uptake under continuous medium flow.

## 5 Conclusion

We demonstrated that a large proportion of CPC-EVs was retained in the mouse heart after intramyocardial injection. Shortly after administration, we observed CPC-EV migration into the interstitial space followed by interaction with cardiac cells. This implies that there is no direct flush-out of EVs from the heart. Whether cardiac retention correlates to CPC-EV function as demonstrated in previous therapeutic studies has yet to be determined. Furthermore, CPC-EV targeting of endothelial cells under continuous medium flow *in vitro* could be enhanced by the expression of RGD-4C and CRPPR targeting peptides on the CPC-EV surface. These insights into the sustained presence of CPC-EVs after cardiac administration that possibly still evoke therapeutic effects, and the possibility to direct them to specific cells present in the heart further expand the application of CPC-EVs in cardiac repair.

## Data Availability

The original contributions presented in the study are included in the article/Supplementary Material, further inquiries can be directed to the corresponding author.
