# Supplementary material for: Evaluation and manipulation of tissue and cellular distribution of cardiac progenitor cell-derived extracellular vesicles
Source: Front Pharmacol. 2022 Nov 24;13:1052091. doi: 10.3389/fphar.2022.1052091 (PMC9729535; doi:10.3389/fphar.2022.1052091)
Supplement: Supplementary file 3 [file DataSheet1.docx]

**Supplemental data**

**Supplementary Figures**

**
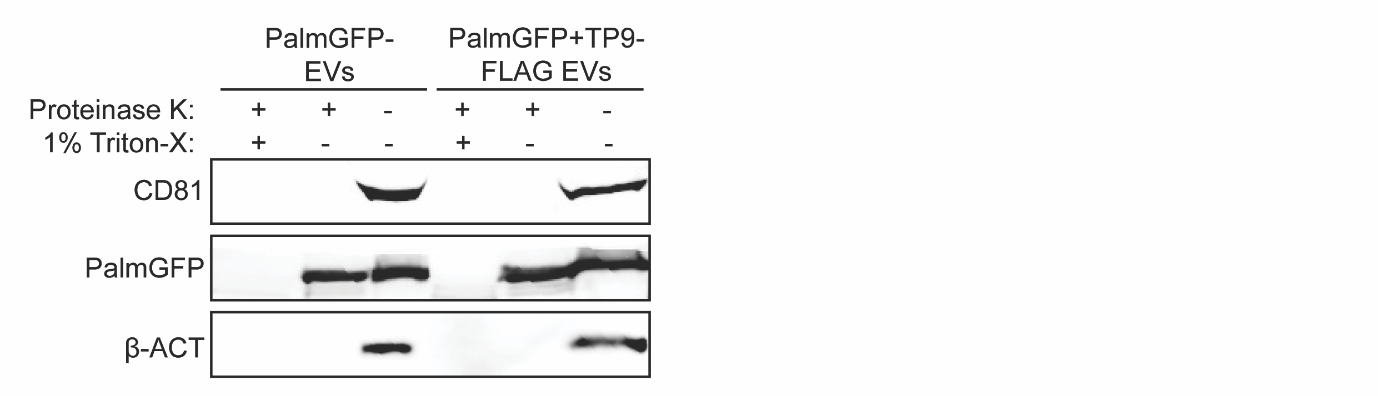
**

**Supplementary Figure S1.** PalmGFP is present in the lumen of EVs. Western blot analysis showing presence or absence of CD81, PalmGFP and ß-actin (ß-ACT) in PalmGFP- and PalmGFP+TP-FLAG expressing EVs after treatment with 1% Triton-X and subsequent Proteinase K.


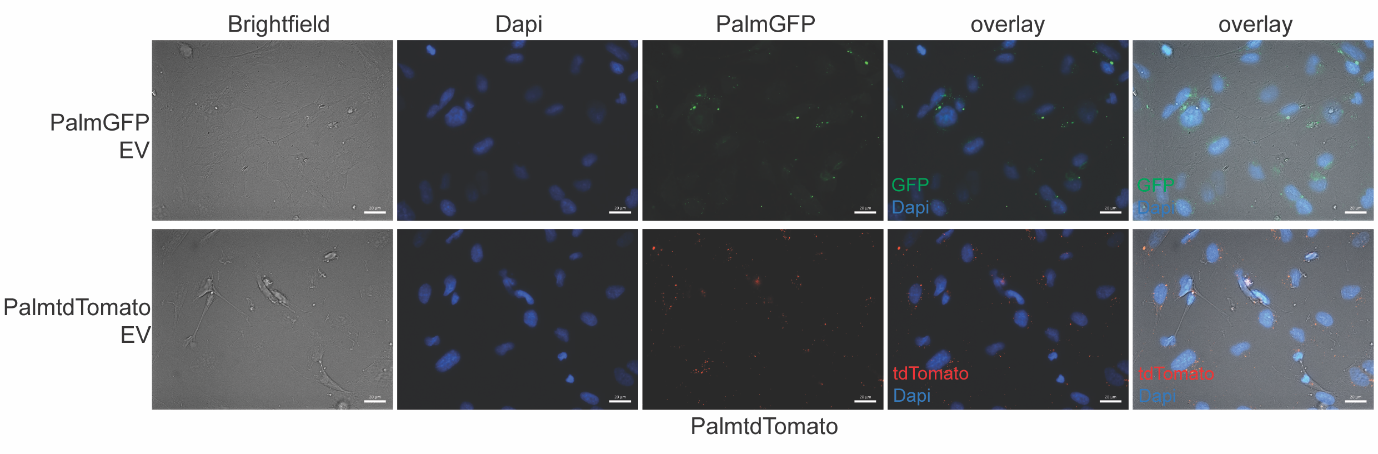


**Supplementary Figure S2.** Uptake of PalmGFP^+^ and PalmtdTomato^+^ EVs in HMEC-1. Fluorescent microscopy pictures of HMEC-1 incubated for 4 hrs with PalmGFP- and PalmTdTomato-labeled EVs, and co-stained with DAPI. Scale bars represent 20 µm.

**
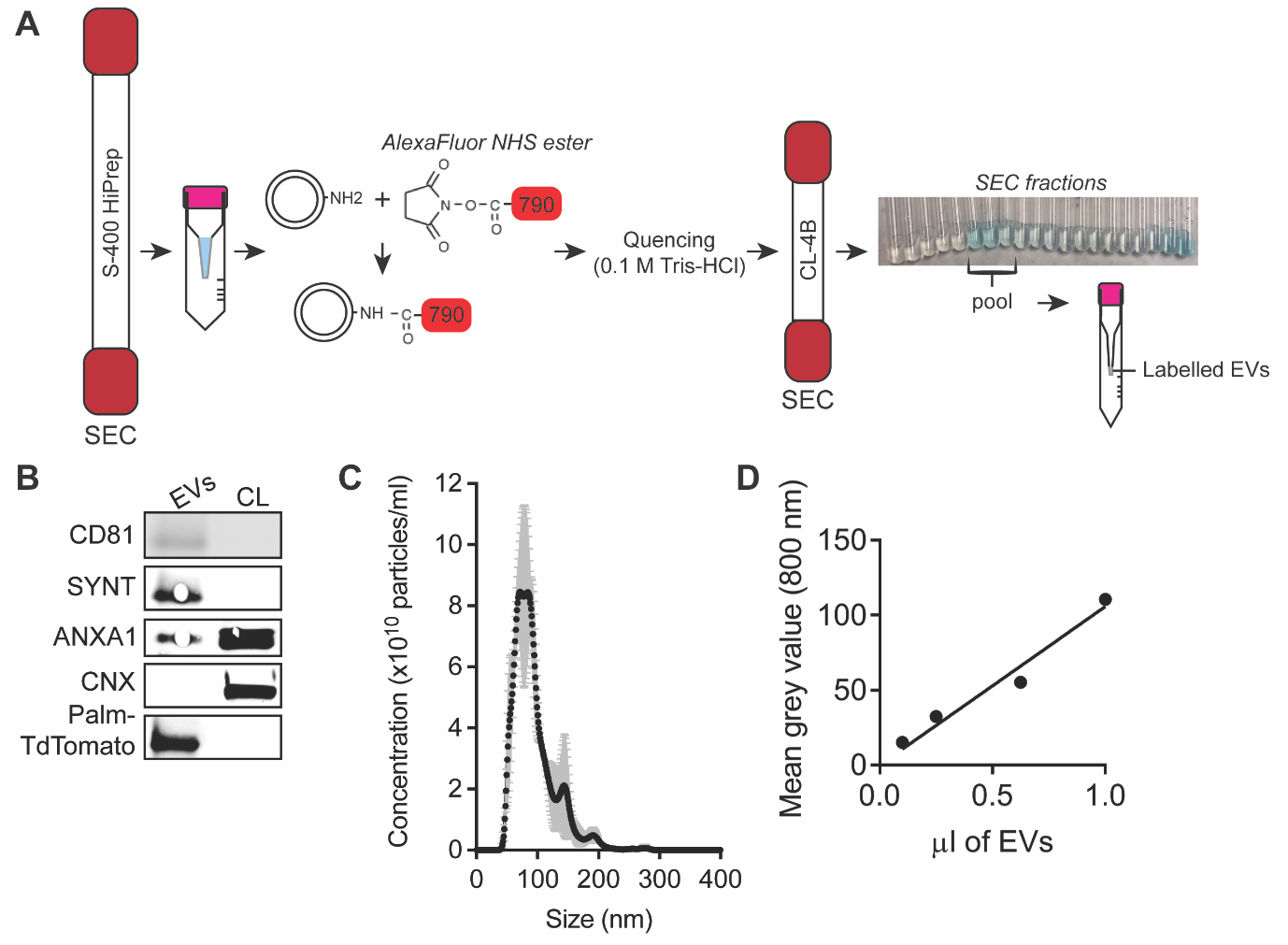
**

**Supplementary Figure S3.** Alexa Fluor NHS ester labeling of PalmTdTomato-EVs. (A) Schematic of (PalmtdTomato^+^) EV isolation by size-exclusion chromatography (SEC), followed by concentrating using 100 kDa molecular weight cut-off spin filters. EVs in small sample volume are labeled with Alexa Fluor NHS ester, quenched with Tris-HCl. Labeled EVs are separated from free dye by consecutive second SEC purification and concentration steps. (B) Western blot analysis showing the presence of CD81, Syntenin-1 (SYNT), AnnexinA1 (ANXA1), Palm-TdTomato, and absence of Calnexin (CNX) in NHS ester-labeled EVs. Cell lysate (CL) derived from control (tdTomato^-^) CPCs was included as control. (C) Representative NTA plot showing the size distribution and particle concentration of Alexa Fluor NHS ester-labelled EVs. (D) Fluorescence in different volume of EVs, measured at 800 nm, determined as mean grey value, corrected for background (PBS).


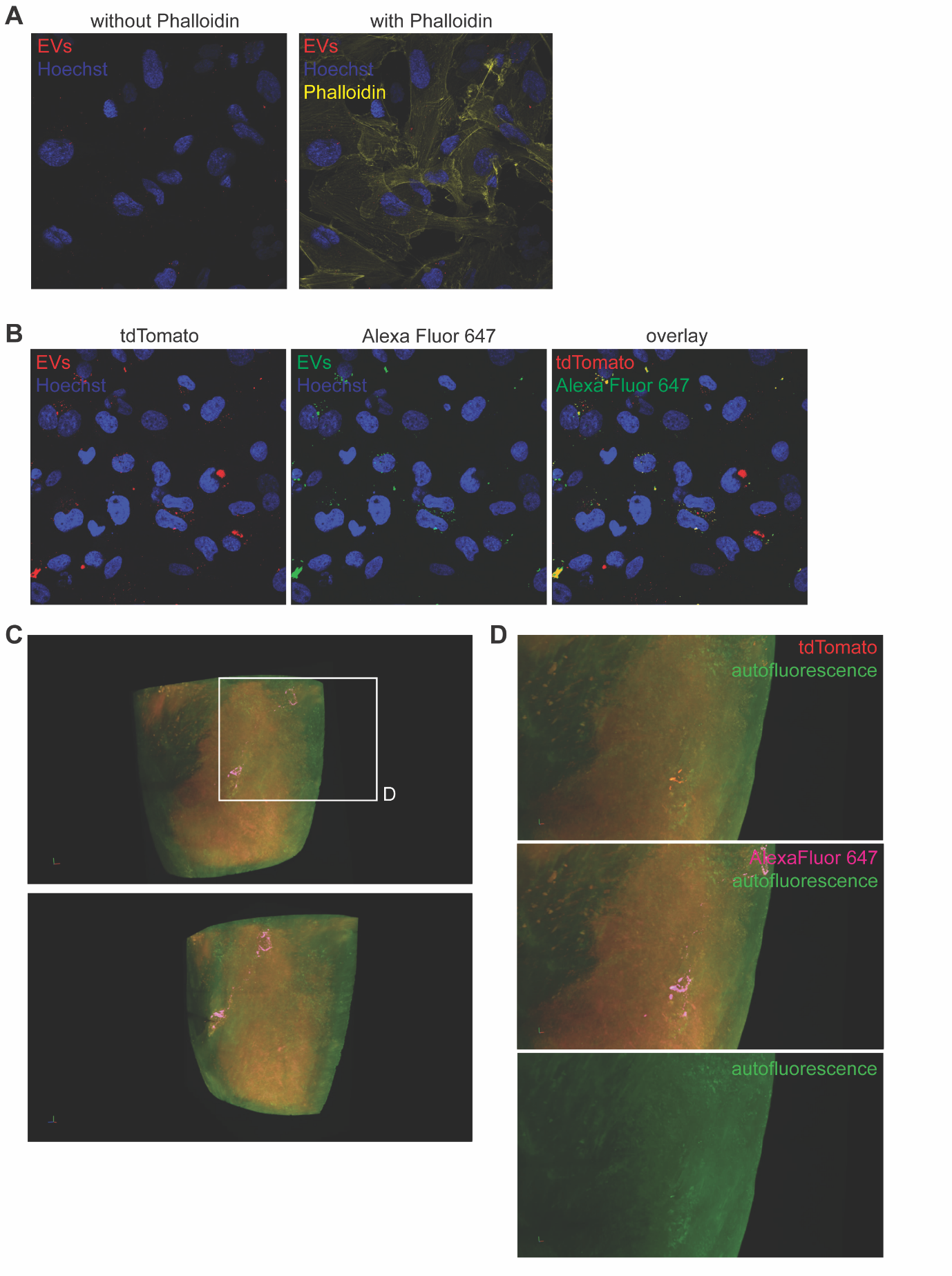


**Supplementary Figure S4.** Uptake of AlexaFluor647 NHS ester-labeled PalmtdTomato^+^ EVs *in vitro* and *in vivo*. (A,B) Fluorescent microscopy pictures of HMEC-1 administered with AlexaFluor 647 NHS ester-labeled PalmtdTomato^+^ EVs after 4 hrs incubation. (A) Cells were co-stained with phalloidin488 and DAPI. (B) Co-localization of PalmtdTomato and AlexaFluor 647 dyes, with DAPI co-stain. (C) Snap-shots of 3D fluorescent images of the heart injected with 1.75x10^9^ AlexaFluor647 NHS ester-labeled TdTomato^+^ EVs, generated after CUBIC tissue clearing and subsequent light-sheet fluorescent microscopy with insert in (D). (D) Fluorescent images with separation of tdTomato and AlexaFluor657 channels.


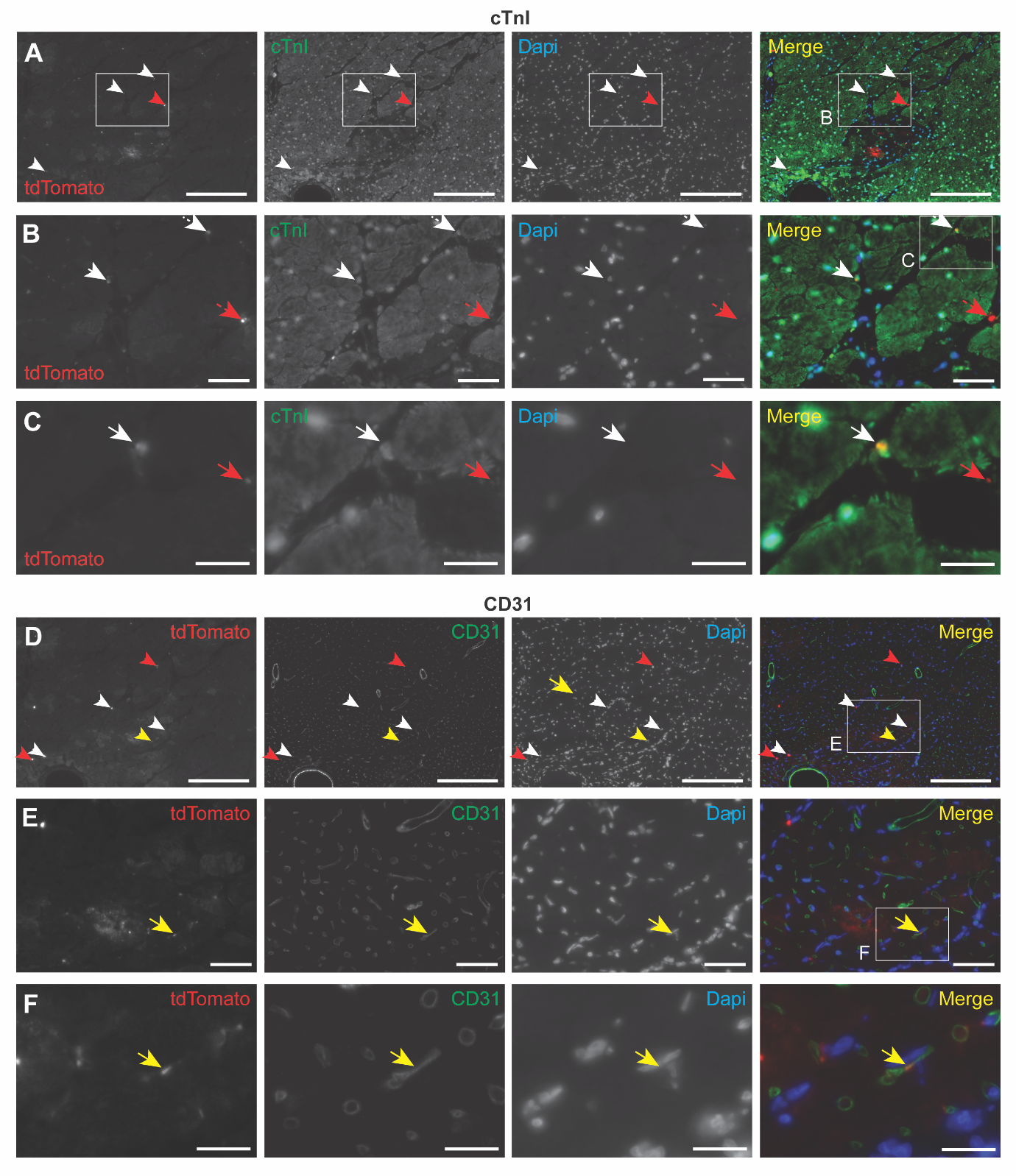


**Supplementary Figure S5.** Immunocytochemistry analysis of EV uptake in the heart. PalmtdTomato-EVs were administered in the left ventricle wall of a healthy mouse heart through intramyocardial injection and heart tissue was collected after 4 hrs. (A-F) Immunofluorescence staining of two subsequent heart sections, cut across the transverse plane, using antibodies against tdTomato (shown in red), and co-staining with antibodies against (A-C) cardiomyocyte marker cardiac Troponin I (cTnI, shown in green) and (D-F) blood vessel-specific CD31 (shown in green). (B) Enlargement of the square in panel A. (C) Enlargement of the square in panel B. (E) Enlargement of the square in panel D. (F) Enlargement of the square in panel E. Nuclei are visualized with DAPI (shown in blue). tdTomato co-localization with other stainings are indicated with arrows: cTnI (white), CD31 (yellow), no co-localization (red). Scale bars = 50 μm (A, D), 250 μm (B, E), 100 μm (C, F).


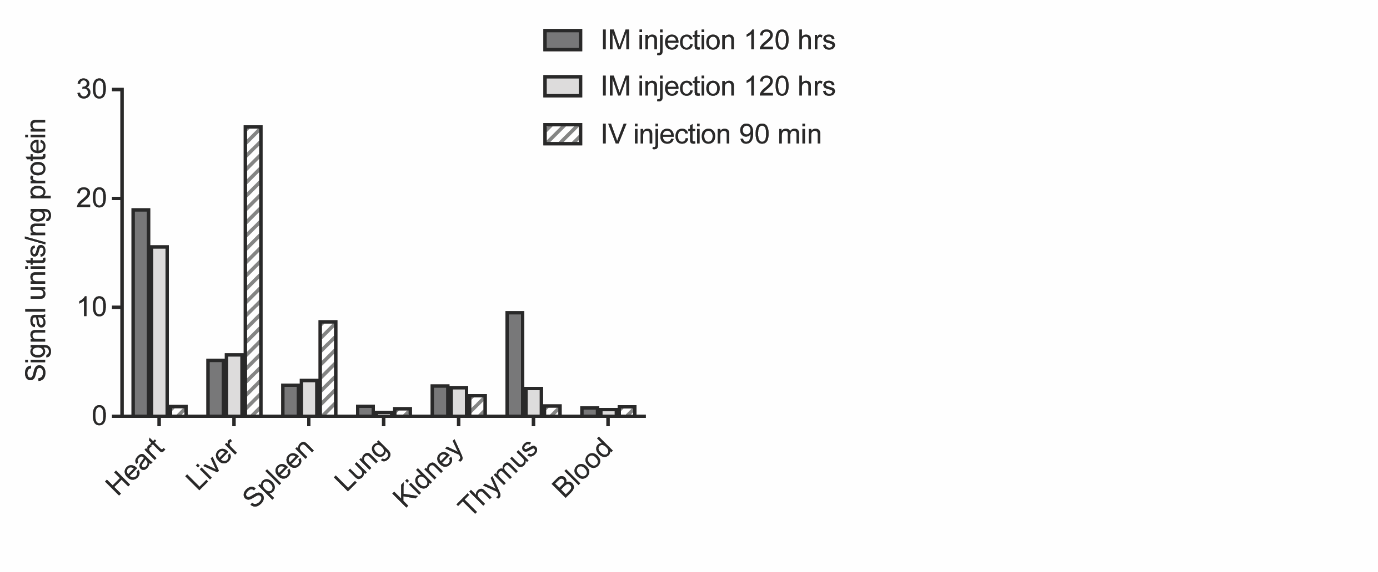


**Supplementary Figure S6.** Organ lysate analysis after intravenous (IV) and intramyocardial (IM) injection of 790NHS ester labeled EVs. Quantification of fluorescence (800nm) per ng protein in organ lysates as compared to organ background. Data are plotted against results derived from intramyocardial injection (organs collected after 5 days follow-up).


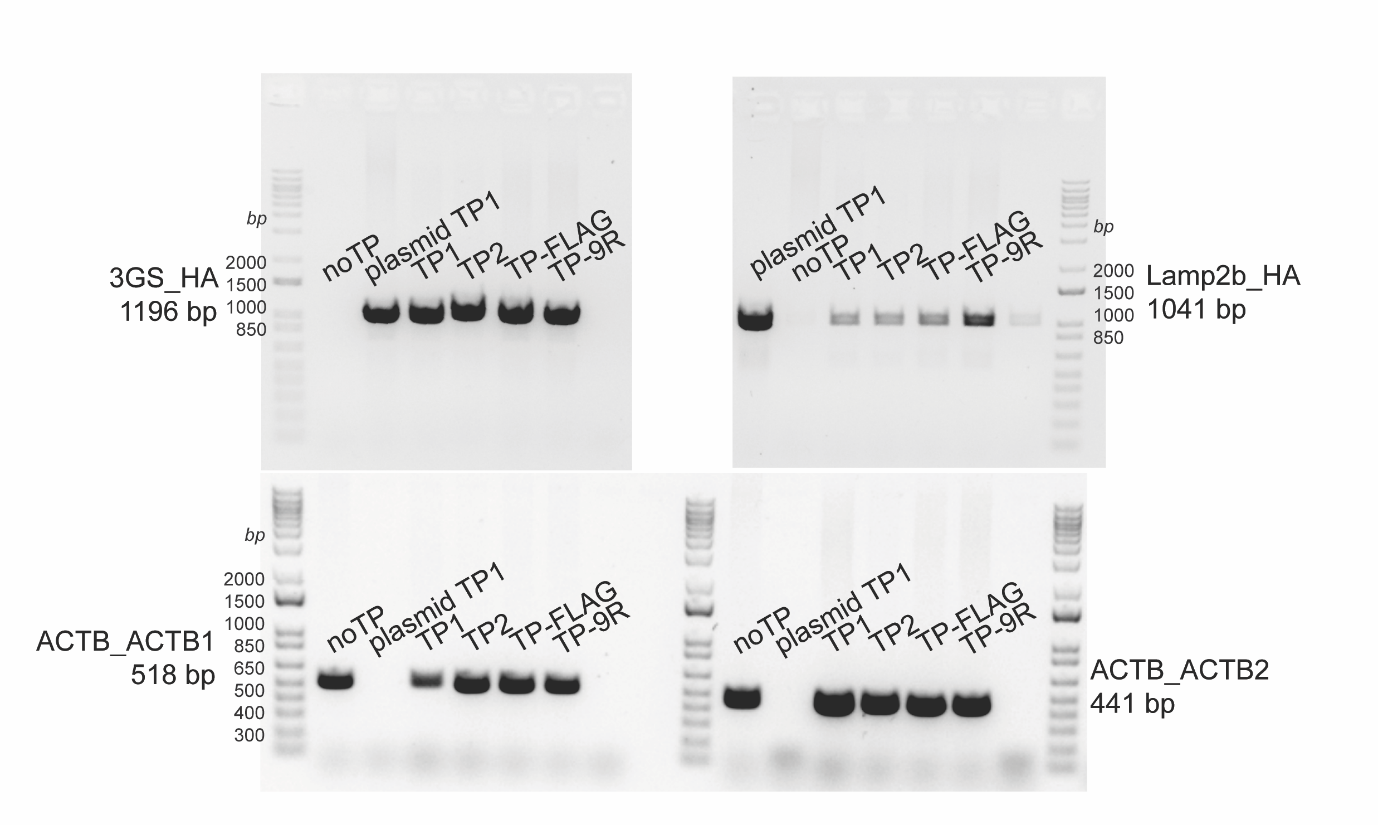


**Supplementary Figure S7.** TP-Lamp2b constructs are incorporated in the CPC genome. PCR amplification of stable CPC lines expressing TPx-Lamp2b-HA constructs. Construct specific primer pairs consist of: 3GS: forward primer in GSG linker region; HA: reverse primer in HA region; Lamp2b: forward primer in Lam2b region. Primer pairs (ACTB1 & ACTB2) for amplification of ACTB were included to control for the presence of genomic DNA. The plasmid expressing TP1 was included as positive control. noTP: genomic DNA of CPCs without construct incorporation. Primer sequences can be found in Supplemental Table 2.


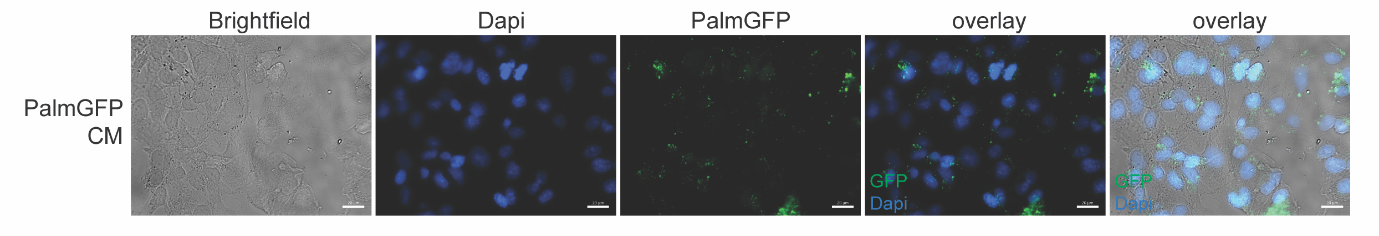


**Supplementary Figure S8.** Uptake of PalmGFP^+^ EV-enriched conditioned medium in HMEC-1. Fluorescent microscopy pictures of HMEC-1 incubated for 4 hrs with PalmGFP^+^ concentrated conditioned medium (CM), and co-stained with DAPI. Scale bars represent 20 µm.


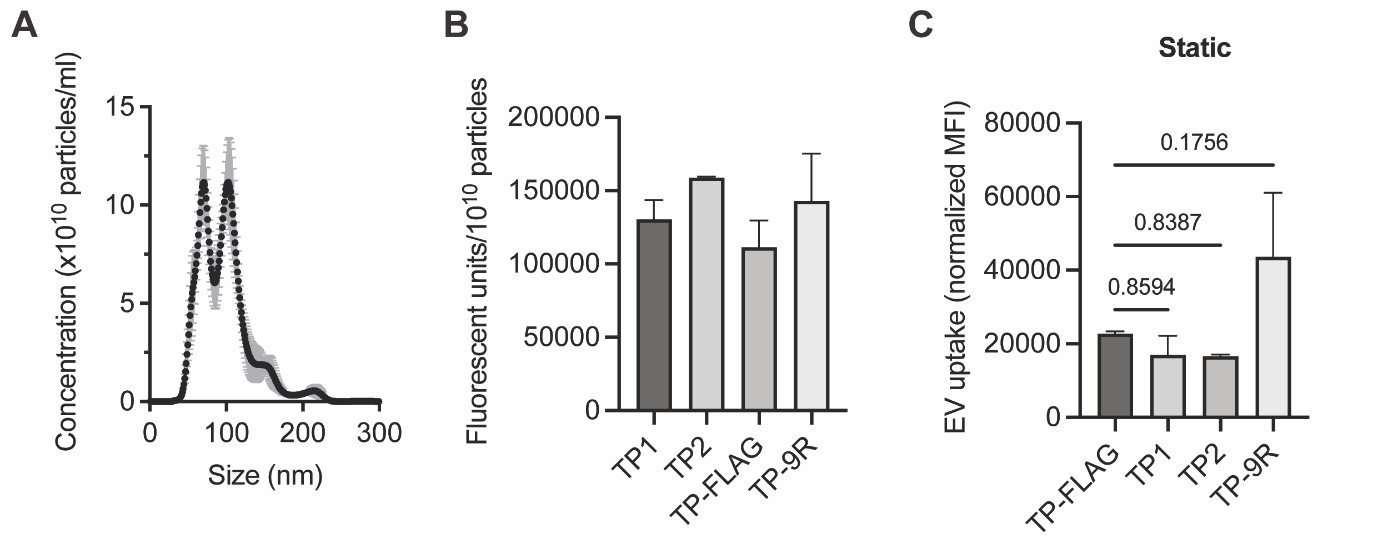


**Supplementary Figure S9.** EV-TPx uptake in HMEC-1 in static conditions. (A) Representative NTA plot showing the size distribution and particle concentration of concentrated conditioned medium (CM) derived from PalmGFP^+^ TP-9R-expressing CPCs. (B) GFP fluorescence per 10^10^ particles determined in CM derived from PalmGFP^+^ TP-expressing CPCs (n=2). Data are displayed as mean ± SD. (C) EV-TP1, -TP2, -TP-FLAG and -TP-9R uptake in HMEC-1 after 4 hrs incubation in static conditions, as determined by flow cytometry. Mean fluorescence is corrected for negative control (M199 medium administration). Data are displayed as mean ± SD (n=2).

**
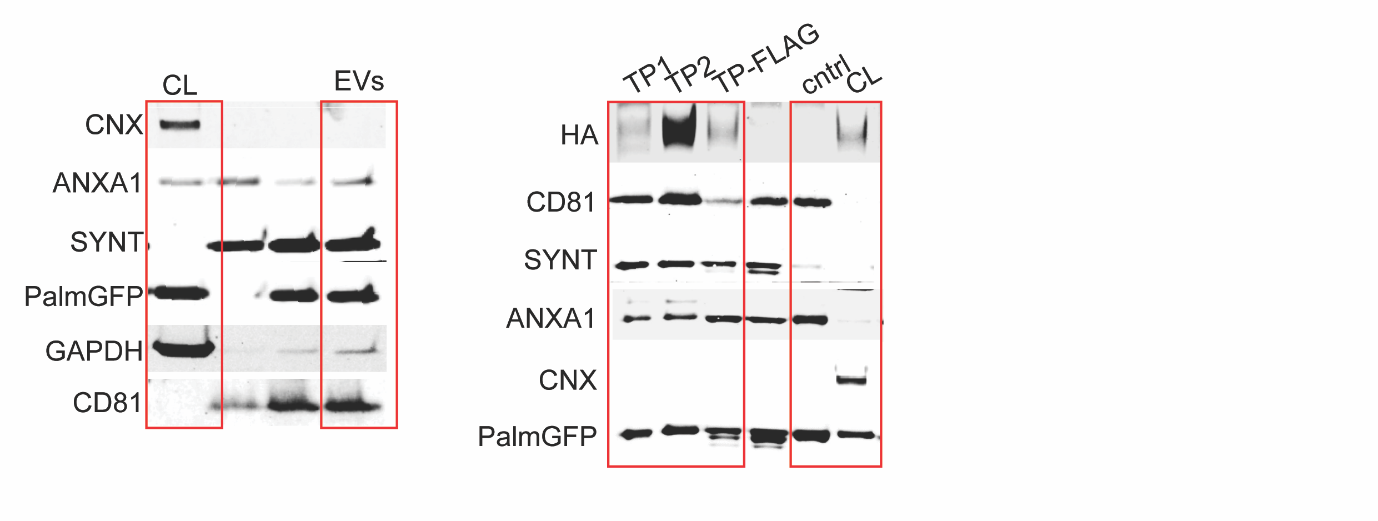
**

**Supplementary Figure S10.** Complete western blots of Figures (left) 1C and (right) 6F.


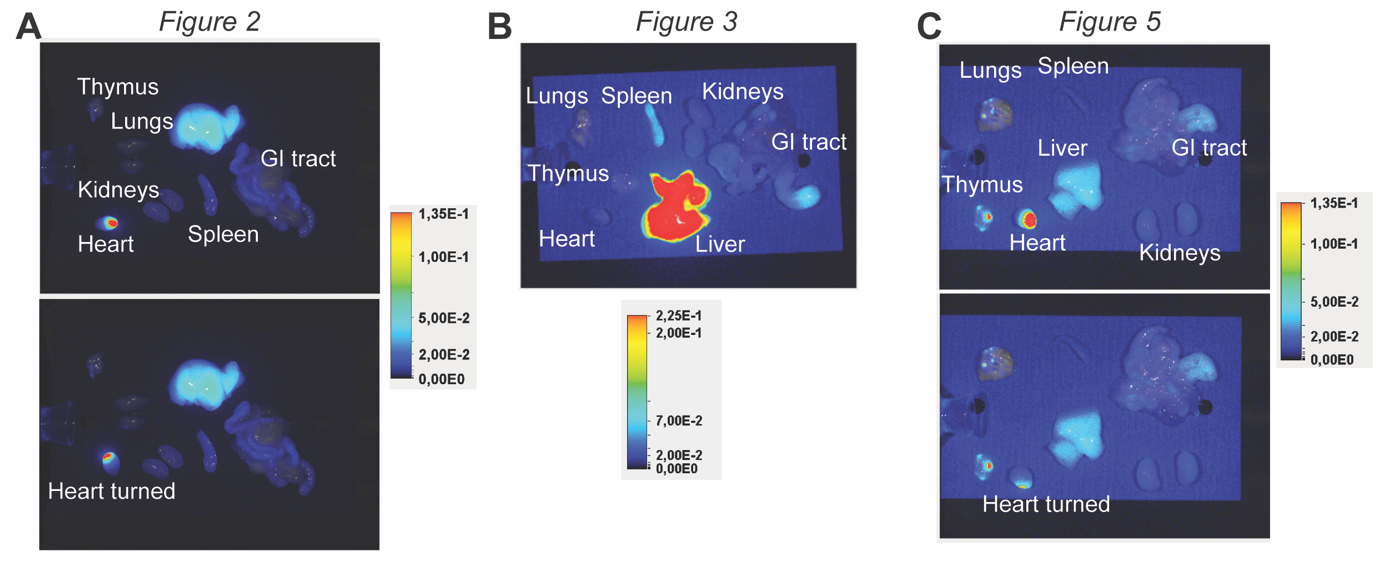


**Supplementary Figure 11.** Uncut NIRF images of individual organs. Figure (A) 2C, (B) 3D, and (C) 5C.

**Supplementary Movie S1.** 3D-reconstruction of the left ventricle injected with 2.5x10^10^ AlexaFluor647 NHS ester-labeled EVs (in red), generated after CUBIC tissue clearing and subsequent light-sheet fluorescent microscopy. Heart was perfused with Lectin-FITC to stain blood vessels (in green) before tissue collection.

**Supplementary Movie S2.** 3D-reconstruction of part of the left ventricle injected with AlexaFluor647 NHS ester-labeled EVs (in red), generated after CUBIC tissue clearing and subsequent light-sheet fluorescent microscopy. Heart was perfused with Lectin-FITC to stain blood vessels (in green) before tissue collection.

| Supplementary Table S1. Protein and nucleotide sequences of targeting peptides (TP) | | | | | | | | | | | | | |
| --- | --- | --- | --- | --- | --- | --- | --- | --- | --- | --- | --- | --- | --- |
|  |  | | |  | |  | | |  | | |  | |
| Name | | Cellular target | | | Amino acid sequence | | | Nucleotide sequence | | |  | |  |
| TP1 | | αvβ1,3,5 integrins | | | ACDCRGDCFCG | | | GCGTGCGATTGCCGCGGCGATTGCTTTTGCGGC | | | F: 5'-**TGGA**GCGTGCGATTGCCGCGGCGATTGCTTTTGCGGC-3' | | R: 5'-**TGCC**GCCGCAAAAGCAATCGCCGCGGCAATCGCACGC-3' |
| TP2 | | Cardiac endothelium | | | CRPPR | | | TGTCGTCCGCCGCGT | | | F: 5'-**TGGA**TGTCGTCCGCCGCGT-3' | | R: 5'-**TGCC**ACGCGGCGGACGACA-3' |
| FLAG | | Negative control | | | DYKDDDDK | | | GACTATAAAGATGACGATGACAAA | | | F: 5'-**TGGA**GACTATAAAGATGACGATGACAAA-3' | | R: 5'-**TGCC**TTTGTCATCGTCATCTTTATAGTC-3' |
| 9R | | Positive control | | | RRRRRRRRR | | | CGTCGCCGACGGAGAAGGCGTCGCCGA | | | F: 5'-**TGGA**CGTCGCCGACGGAGAAGGCGTCGCCGA-3' | | R: 5'-**TGCC**TCGGCGACGCCTTCTCCGTCGGCGACG-3' |
|  | | |  | | | |  | | | **BbsI** restriction site overhangs | | | |

**Supplementary Tables**

| Supplementary Table S2. Primer sequences of TP-Lamp2b-HA construct detection | | | |
| --- | --- | --- | --- |
|  |  | *Detection* | *Amplicon size* |
| 3GS_HA | F: 5'-TCCGGTGGCTCGAGTTTGG-3' | Construct |  |
|  | R: 5'-AGCGTAATCTGGCACATCGTA-3' |  | 1196 |
| Lamp2b_HA | F: 5'-TGGAAGCATTTGTGGGGATGA | Construct |  |
|  | R: 5'-AGCGTAATCTGGCACATCGTA-3' |  | 1041 |
| ACTB_ACTB1 | F: 5'-CATGTACGTTGCTATCCAGGC-3' | ACTB genomic DNA |  |
|  | R: 5'-ACAGGACTCCATGCCTGAGAG-3' |  | 518 |
| ACTB_ACTB2 | F: 5'-TGCTATCCCTGTACGCCTCTG-3' | ACTB genomic DNA |  |
|  | R: 5'-GGACTTAGCTTCCACAGCACAG-3' |  | 441 |
